# Intratumoral delivery of immunotherapy to treat breast cancer: current development in clinical and preclinical studies

**DOI:** 10.3389/fimmu.2024.1385484

**Published:** 2024-05-13

**Authors:** Siena M. Mantooth, Yara Abdou, Ana Rosa Saez-Ibañez, Samik Upadhaya, David A. Zaharoff

**Affiliations:** ^1^Joint Department of Biomedical Engineering, North Carolina State University and University of North Carolina at Chapel Hill, Raleigh, NC, United States; ^2^Lineberger Comprehensive Cancer Center, University of North Carolina at Chapel Hill, Chapel Hill, NC, United States; ^3^Cancer Research Institute, New York, NY, United States

**Keywords:** breast cancer, cancer immunotherapy, intratumoral, localized delivery, neoadjuvant, hydrogel, delivery systems

## Abstract

Breast cancer poses one of the largest threats to women’s health. Treatment continues to improve for all the subtypes of breast cancer, but some subtypes, such as triple negative breast cancer, still present a significant treatment challenge. Additionally, metastasis and local recurrence are two prevalent problems in breast cancer treatment. A newer type of therapy, immunotherapy, may offer alternatives to traditional treatments for difficult-to-treat subtypes. Immunotherapy engages the host’s immune system to eradicate disease, with the potential to induce long-lasting, durable responses. However, systemic immunotherapy is only approved in a limited number of indications, and it benefits only a minority of patients. Furthermore, immune related toxicities following systemic administration of potent immunomodulators limit dosing and, consequently, efficacy. To address these safety considerations and improve treatment efficacy, interest in local delivery at the site of the tumor has increased. Numerous intratumorally delivered immunotherapeutics have been and are being explored clinically and preclinically, including monoclonal antibodies, cellular therapies, viruses, nucleic acids, cytokines, innate immune agonists, and bacteria. This review summarizes the current and past intratumoral immunotherapy clinical landscape in breast cancer as well as current progress that has been made in preclinical studies, with a focus on delivery parameters and considerations.

## 1 Introduction

Breast cancer (BC) is the most common cancer among women, with about 2.3 million new cases diagnosed globally in 2020 and 3 million new cases projected in the year 2040 (1). The BC five-year survival rate, according to the Surveillance, Epidemiology, and End Results (SEER) database, is 90% for all BC types and grades. Local and distant recurrence present one of the great problems in BC treatment. Metastases are responsible for >90% of BC deaths, and the 5-year relative survival rate for women with metastatic BC in the U.S. is 30%, according to SEER. However, only 6% of BC patients are initially diagnosed with stage IV disease (2–4). Thus, the majority of deaths are due to localized disease that progressed and metastasized after initial treatment. Despite great progress with diagnosis and treatment of BC, metastatic disease remains incurable. Some subtypes have higher likelihood of metastasis, with triple negative BC (TNBC) and human epidermal growth factor receptor 2 (HER2)-enriched tumors being more likely to recur within 5 years of treatment compared to the luminal subtypes (4, 5).

Immunotherapy, treatments to engage the immune system, has gained interest in the last decade for cancer treatment. The promise of immunotherapy is that it has the potential to generate durable cures, thus raising the tail of the survival curve (6, 7). The monoclonal antibody anti-programmed cell death protein 1 (aPD-1) checkpoint inhibitor pembrolizumab is an approved immunotherapy for unresectable locally advanced or metastatic TNBC (8) as well as a neoadjuvant and adjuvant for early-stage, high risk TNBC (9). Other immunotherapeutics, such as viruses, cells, cytokines, and innate immune agonists, are being or have been evaluated in clinical studies (10, 11). As of 2022, 778 total agents were actively being developed for use in breast cancer and TNBC, at various developmental stages (11). Rationally delivered immunotherapies can stimulate the immune system for lasting, durable responses. Furthermore, tumor infiltrating lymphocytes (TILs) have been demonstrated to be a positive biomarker for improved therapeutic efficacy in BC (12), and delivery of immunotherapeutics has the potential to increase TILs and other immune cells to generate robust anti-tumor activity.

However, immunotherapeutics delivered systemically can also lead to severe adverse events (AEs). For example, the use of checkpoint inhibitors in BC patients has led to serious immune related AEs, with the most frequently reported being rash (38%), infusion reaction (12%), and hypothyroidism (12%) (13–16). These toxicities limit the utilization of these drugs, and therefore, ways to optimize treatment delivery while minimizing toxicity are urgently needed.

One solution to address these challenges is to deliver immunotherapeutics locally at the site of the tumor as an intratumoral (i.t.) injection. I.t. delivery increases therapeutic concentration locally, which could improve treatment efficacy and reduce systemic AEs. Additionally, certain immunotherapeutics can locally stimulate an immune response at the site of the tumor as an *in situ* vaccine (17). The FDA-approval of i.t. oncolytic herpes virus talimogene laherparepvec (T-VEC) in metastatic melanoma demonstrates the potential for localized immunotherapy to induce systemic antitumor immunity (18).

Additionally, BC tumors typically present with an immunosuppressive tumor microenvironment, infiltrated by a host of immunosuppressive cells such as regulatory T cells (Tregs), myeloid-derived suppressor cells (MDSCs), and tumor associated macrophages (TAMs) (19). Thus, historically, BC has been considered an immunologically cold tumor, with few anti-tumor effector immune cells, rendering systemic immunotherapy ineffective for a majority of patients (20, 21). I.t. delivered immunotherapy could directly recruit effector immune cells into the tumor microenvironment while antagonizing and reducing immunosuppressive cells. When developing i.t. immunotherapies, numerous considerations should be taken into account. Delivery mediums, including but not limited to hydrogels, microparticles, and nanoparticles (NPs), can be engineered to improve retention within the tumor and prolong release locally. Timing of delivery relative to prior or concomitant treatments can also impact outcomes. For instance, neoadjuvant immunotherapy can reduce tumor burden prior to resection, and it may additionally be able to re-educate the immune system using tumor-specific, patient-specific antigens. Also, the frequency of treatment should be considered, as patients can have local pain and discomfort from i.t. injections (22).

**Figure 1:**
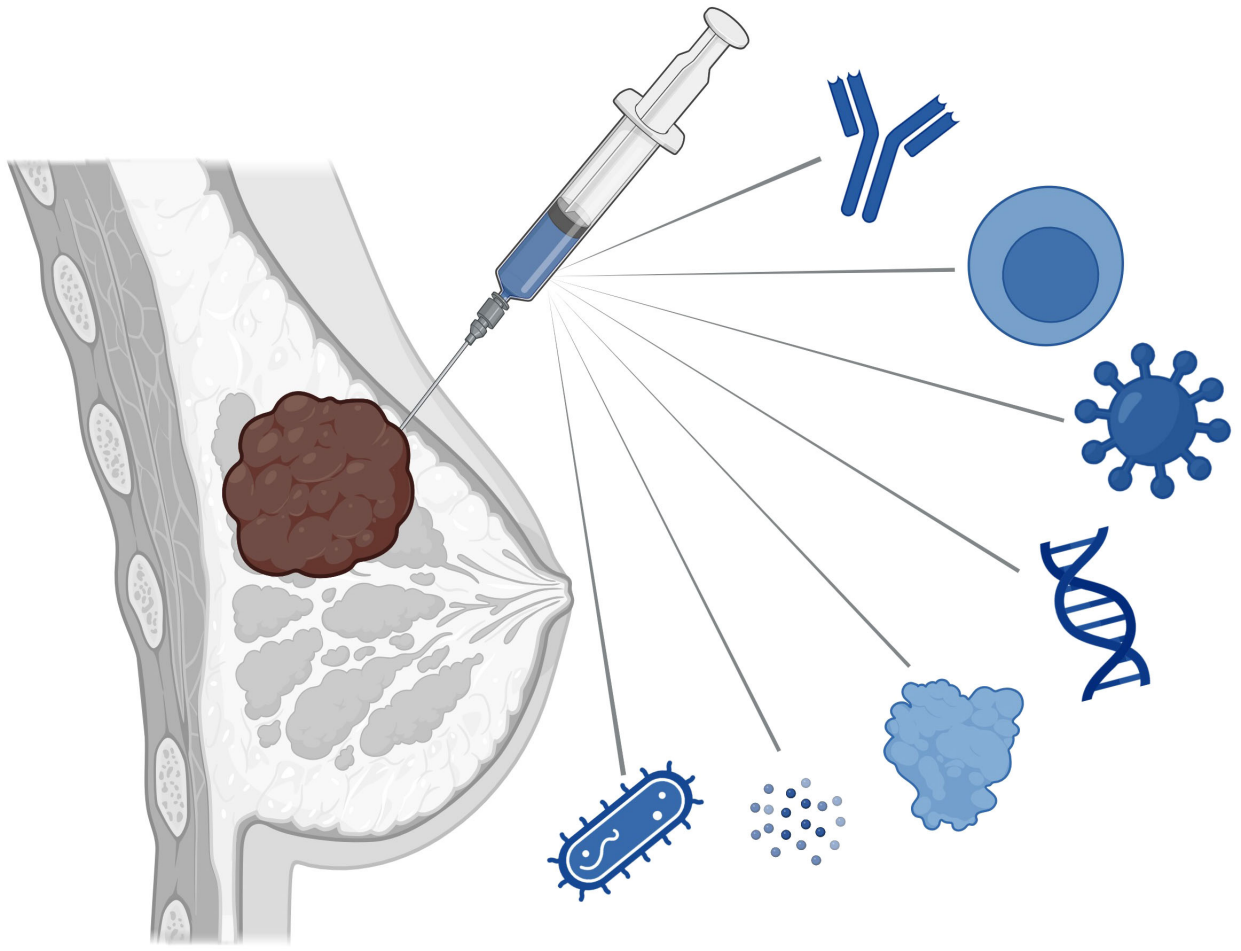
Intratumoral immunotherapies being explored in clinical and preclinical studies in breast cancer treatment include monoclonal antibodies, cellular therapies, viruses, nucleic acids, cytokines, innate immune agonists, and bacteria.

In this review, we will present findings from clinical and preclinical BC studies that have evaluated an i.t. immunotherapy (**Figure 1**). Section II will introduce the different types of immunotherapies, providing a historical context and mechanisms of action. Section III will present completed and ongoing clinical trials of i.t. delivered immunotherapeutics for BC, highlighting the types, timing, and outcomes of treatment. Section IV will present preclinical strategies, with a focus on immunotherapy types, delivery timing and frequency, and delivery mediums. Section V will summarize key findings from the review. Section VI will reflect on future steps for the field of i.t. delivered BC immunotherapies.

## 2 A historical perspective of types of immunotherapy in clinical and preclinical BC studies and their mechanisms

The following section will introduce the history and mechanism of seven technologies that have been studied in the context of BC, including checkpoint inhibitors and other monoclonal antibodies, cellular therapies, viruses, nucleic acids, cytokines/chemokines, innate immune agonists, and bacteria. Thus far, the only clinically approved immunotherapeutic for BC is the aPD-1 checkpoint inhibitor pembrolizumab (Keytruda) for TNBC. The remaining technologies are in clinical and/or preclinical development.

### 2.1 Checkpoint inhibitors and other monoclonal antibodies

In the 1990’s, researchers Professor Tasuku Honjo and Professor James Allison discovered the cell surface receptors programmed death protein-1 (PD-1) and cytotoxic T lymphocyte-associated antigen 4 (CTLA-4), respectively, and their roles on the suppression of T cell activity. Honjo and Allison shared the Nobel Prize in Medicine in 2018 for their discovery that inhibition of PD-1 and CTLA-4 signaling can suppress negative immune regulation and provide a new form of cancer therapy (23). Today, aPD-(L)1 and aCTLA-4 have been approved for certain cancer indications in the clinic, and in particular, the aPD-1 checkpoint inhibitor pembrolizumab has been approved for certain BC indications (24). A next-generation checkpoint inhibitor antibody against lymphocyte activation gene-3 (LAG-3) was recently approved for a non-BC cancer indication (25). Other next-generation checkpoint inhibitors currently in development include antibodies against T cell immunoglobulin-3 (TIM-3), B7-H3 and B7-H4, A2aR and CD73, natural killer group protein 2A, and poliovirus receptor-related immunoglobulin domain containing (26, 27). Other monoclonal antibodies, which have not yet received regulatory approval, bind to costimulatory molecules, such as OX40, which enhances T cell receptor signaling (28) and CD40, which activates dendritic cells (DCs) (29).

### 2.2 Cells

Cellular therapies utilize either autologous or allogeneic cells, derived from non-stem and stem cells, which may or may not be genetically engineered or manipulated (30). The first recorded instance of cellular therapy was in the 19^th^ century by Charles-Édouard Brown-Séquard who utilized extracts of animal testicles to prevent aging (31). In cancer therapy, chimeric antigen receptor (CAR) T cells, in which autologous T cells are genetically engineered to express a CAR directed against a particular target and kill tumor cells in a non-MHC-dependent manner, have demonstrated significant clinical success in blood tumors, and numerous efforts are being made to improve their efficacy in solid tumors. Other T cell immunotherapies utilize injections of activated, *ex vivo* differentiated and expanded allogenic T cells (AlloStim) to serve as an *in situ* cancer vaccine by evoking an immune response (32, 33). Another vaccination-based approach uses DCs to serve as a bridge between innate and adaptive immunity with the ability to present antigens to T cells. A variety of approaches have utilized DCs in cancer immunotherapy, the most common of which being *ex vivo* derived DCs loaded with tumor antigens (34). Finally, other less commonly utilized cellular products include other immune cells, such as natural killer (NK) cells and macrophages as well as fibroblasts, which can be engineered for enhanced anti-tumor immunity (35).

### 2.3 Viruses

In the early 1900s, viruses were observed to improve the outcome of cancer treatment, and in the mid 1900’s, reports of certain wild type viruses provided variable benefit in cancer patients (36). Many of these viruses were not consistently effective, but adenoviruses demonstrated acceptable safety with potential to induce necrosis in cervical cancers (36). Oncolytic viruses, which specifically target and lyse cancer cells, have entered clinical trials, and T-VEC, a herpes virus also carrying the gene for cytokine granulocyte-macrophage colony-stimulating factor (GM-CSF), is approved for the local treatment of unresectable metastatic melanoma (36, 37). Numerous oncolytic viruses are in preclinical and clinical development, including but not limited to adenoviruses, Newcastle disease viruses, herpes viruses, and measles viruses (38). Though some wild type viruses have clinical utility, oncolytic viruses can be engineered to enhance their anti-tumor action through confining viral replication only to cancer cells, targeting receptors that are more highly expressed on cancer cells, and incorporating transgenes for other anti-tumor biologic cargo (36). Other viral based techniques also utilize the ability of viruses to infect a cell as a gene delivery vehicle for intracellular delivery or to stimulate the immune system as an immune agonist.

### 2.4 Nucleic acids

In addition to viral mediated gene delivery, other nucleic acid-based deliveries include plasmid and mRNA-based delivery. Plasmids are small, circular structures of DNA, and for cancer immunotherapy, they can be engineered to encode tumor antigens and immunomodulatory or cytotoxic proteins. Plasmids can be delivered alone, with the assistance of physical forces, such as through sonication or electric pulses, or complexed with lipids, polymers, or other compounds for enhanced therapeutic efficacy (39). Plasmids are cost-effective, easy to manufacture, and demonstrate high stability. Poor delivery to the nucleus where plasmid-encoded genes are transcribed limits the efficacy of plasmid-based immunotherapies (39). On the other hand, mRNA therapeutics, which have recently gained public attention with SARS-CoV-2 vaccines, need only to be delivered into the cytoplasm of the cell for the transcription and translation of its encoded genetic material. Though less stable than DNA, mRNA can be encapsulated in polymer and/or lipid nano- and micro-particles for enhanced stability and transfection.

### 2.5 Cytokines and chemokines

Cytokine and chemokine immunotherapies seek to modulate the tumor microenvironment (TME) from a tumor supporting, immune suppressive milieu to a tumor attacking, immune activated one. Cytokines are small proteins, often below 30kDa, which signal cell activation, growth, differentiation, and apoptosis (40). Cytokines serve as key modulators of the immune environment, and certain pro-inflammatory cytokines possess anti-tumor activity, which has led to the clinical approval of interleukin-2 (IL-2) and interferon-alpha (IFNα) for certain cancer indications (40). Granulocyte colony-stimulating factor (G-CSF), GM-CSF, vascular endothelial growth factor (VEGF), IL-12, and IFNγ have received the most attention in clinical trials, perhaps due to their relatively earlier discovery compared to the other cytokines, and many others are in clinical development (41). Notably, however, cytokines delivered systemically have a short half-life and can induce profound toxicities, including cytokine release syndrome, which can be fatal (42).

Chemokines are a class of cytokine that drive the chemotaxis or movement of immune cells to a particular site. Chemokines can serve pro- or anti-tumor functions, with the chemokines CXCL9 and CXCL10, ligands for CXCR3, being of the most interest in the anti-tumor category due to their ability to attract effector CD8+ and CD4+ T cells (43). No chemokines are currently clinically approved for therapeutic application.

### 2.6 Innate immune agonists

Innate immune agonists offer another route to stimulate and recruit immune cells, thus altering the TME. Innate immune cells are equipped with pattern recognition receptors (PRRs), which recognize pathogen-associated molecular patterns (PAMPs) and damage-associated molecular patterns (DAMPs). Binding to these PRRs on innate immune cells can elicit an anti-tumor immune response through the induced production of cytokines and immune cell and environment repolarization. PRRs include the membrane-bound toll-like receptors (TLRs) and C-type lectin receptors (CLRs), as well as the cytosolic proteins retinoic acid-inducible gene- (RIG-I)-like receptors (RLRs), AIM2-like receptors (ALRs), NOD-like receptors (NLRs), and stimulator of interferon genes (STING) (44).

### 2.7 Bacteria

Bacteria also can be utilized as a cancer treatment, and the use of such dates back to 1863, in which a patient was intentionally infected with *Streptococcus pyogenes*. Though the tumor was reduced, the patient died from the infection (45). This and other trials with bacteria led to the physician William Coley injecting heat-inactivated *Serratia marcescens* and *Streptococcus pyogenes* into sarcoma, beginning a new field, now known as cancer immunotherapy (45). Localized bacteriotherapy can both serve to initiate an inflammatory response and to colonize the tumor itself, thereby leading to necrosis and cell lysis (46). In particular, the attenuated anaerobic bacterium *Clostridium novyi-NT* has locally initiated necrosis and cell lysis, leading to long-term cellular anti-tumor immunity (46).

## 3 Clinical studies of intratumorally delivered immunotherapeutics for BC

### 3.1 Completed clinical trials

**Figure 2:**
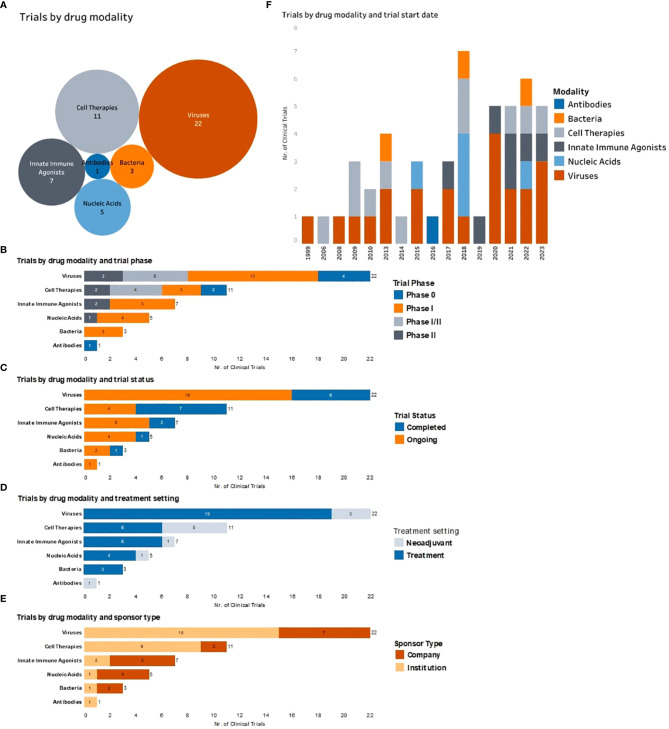
Complete and ongoing clinical studies of intratumorally delivered immunotherapies in breast cancer. **(A)** Bubble diagram representing trials by drug modality. Each bubble represents a different therapeutic modality. The size of each bubble corresponds to the number of trials within that group, also indicated by number. **(B)** Number of clinical trials by therapeutic modality being tested (y axe) and color-coded according to the trial phase. **(C)** Number of clinical trials by therapeutic modality being tested (y axe) and color-coded according to the trial status. **(D)** Number of trials by therapeutic modality being tested (y axe) and color-coded according to the treatment setting (equivalent to treatment timing). **(E)** Number of clinical trials by therapeutic modality being tested (y axe) and color-coded according to the type of trial sponsor. **(F)** Clinical trials organized by trial start year (x axe) and color-coded by type of therapeutic modality being tested.

As of January 2024, seventeen clinical trials successfully completed an evaluation of i.t. delivery of immunotherapeutics in BC. Clinical trials were sourced from clinicaltrials.gov and the Cancer Research Institute’s (CRI) Immuno-Oncology (IO) Intelligence database (**Table 1**, **Figure 2**). For clinical trial sourcing from clinicaltrials.gov, the key search terms used were “breast cancer” AND “intratumoral” OR “intralesional”. Trials were subsequently filtered to include immunotherapy results only. Additional clinical trials were sourced from the CRI’s IO Intelligence database, which is built from GlobalData’s Oncology Trial Database and subsequently curated by CRI based on CRI IO Analytics definition of different immunotherapy types and drug target information. For this publication, immunotherapy trials in which at least one form of BC was listed among the clinical trial indications were included and subsequently filtered by the appearance of the keywords “intratumoral” or “intralesional” in the trial title. Data cut date was January 26, 2024. Limitations to the trial sourcing methods included the impossibility of identifying trials involving treatments administered intratumorally if this was not clearly stated in the trial title.

**Table 1:** Completed clinical trials of intratumoral therapies for breast cancer (clinicaltrials.gov, key search terms “breast cancer” AND “intratumoral” OR “intralesional”) as of January 2024.

Intratumoral Intervention	Description	BC Indication	NCT Number	Phase	Enrollment	Results
Cells						
AlloStim-7	activated, allogenic Th1-like cells; *with cryoablation*	Metastatic	NCT00861107	1/2	50	No Results Posted
AlloStimTM	activated, allogenic Th1-like cells; *with cryoablation*	Stage II-IV	NCT01065441	1/2	9	No Results Posted
anti-HER2 DC1	HER2 peptide-pulsed autologous DC vaccine [neoadjuvant]	HER2+ DCIS	NCT02061332	1/2	58	Has Results (47)
autologous CD1c myeloid DCs	autologous CD1c (BDCA-1)+ myeloid dendritic cells; *with i.t. ipilimumab and avelumab and i.v. nivolumab*	Advanced TNBC	NCT03707808	1b	9	Has Results (48)
autologous DCs	autologous DCs [neoadjuvant]; *with chemotherapy, with or without radiotherapy*	Stage II/III HER2-	NCT00499083	2	17	Has Limited Results
cyclin B1/WT-1/CEF DC	cyclin B1/WT-1/CEF (antigen)-loaded DC vaccination [neoadjuvant]; *with chemotherapy*	Locally advanced TNBC	NCT02018458	1/2	10	Has Results (49)
mRNA c-Met-CAR T cells	chimeric antigen receptor against hepatocyte growth factor receptor (c-Met) T cells	Metastatic and/or TNBC	NCT01837602	1	6	Has Results (50)
Viruses						
Ad5CMV-p53 gene	adenovirus-mediated p53	Metastatic	NCT00004038	1	20	No Results Posted
ADV-hIL12	adenovirus-mediated interleukin-12	Metastatic	NCT00849459	1	3	No Results Posted
Ad-RTS-hIL-12	nonreplicating adenoviral vector for interleukin-12; *with veledimex*	Advanced and/or Metastatic	NCT02423902	1/2	9	Has Results (51)
Ad-RTS-hIL-12	nonreplicating adenoviral vector for interleukin-12; *with veledimex*	Recurrent/Metastatic	NCT01703754	2	12	No Results Posted
HF10	Oncolytic herpes simplex virus 1	Metastatic	NCT01017185	1	28	Has Results (52)
MV-NIS	oncolytic measles virus encoding thyroidal sodium iodide symporter (NIS)	Metastatic	NCT01846091	1	12	Has Results (53)
Nucleic Acids						
IT-pIL12-EP	intratumoral plasmid IL-12 electroporation	Metastatic TNBC	NCT02531425	1	10	Has Results (54)
Innate Immune Agonists						
E7766	STING agonist	Advanced	NCT04144140	1	24	No Results Posted
IMO-2125	TLR9 agonist	Advanced	NCT03052205	1	54	Has Results (55, 56)
Bacteria						
*Clostridium novyi-NT*	attenuated anaerobic bacterium	Advanced	NCT01924689	1	24	Has Results (57)

#### 3.1.1 Cells

Three clinical trials have evaluated DC-based vaccines in a neoadjuvant setting (NCT02061332, NCT02018458, NCT00499083). In a phase 1/2 trial, patients with HER2 overexpressing ductal carcinoma *in situ* (DCIS) or early invasive BC received a HER2-pulsed DC vaccine via intranodal and/or intralesional injection prior to surgery (NCT02061332). The treatment was determined to be safe with only grade 1 and grade 2 AEs reported. Tumor-specific T-cell responses as measured via systemic anti-HER2 CD4+ and CD8+ T cells pre- and post-treatment were increased with vaccination. The route of administration did not impact the immune or clinical response. Pathological complete response (pCR) was higher in patients with DCIS (28.6%) rather than invasive BC (8.3%). In patients with DCIS, the sentinel lymph node (LN) anti-HER2 CD4+ T cell response rather than peripheral blood anti-HER2 CD4+ or CD8+ T cell immune response was associated with pCR (47).

In a second phase 1/2 trial, patients with locally advanced TNBC received a neoadjuvant antigen-loaded DC vaccine concurrently with standard chemotherapy (doxorubicin/cyclophosphamide, then paclitaxel and carboplatin) (NCT02018458). The antigens selected for loading were overexpressed in TNBC, namely cyclin B1 and WT1, along with control viral antigens, CEF (cytomegalovirus, Epstein-Barr virus, and influenza virus) (49). Vaccines were administered i.t. four times pre- and s.c. thrice post- surgery. Patients had grade 1-2 AEs at the injection site (49). 5/10 patients had a pCR at surgery, while 3/10 still had macroscopic residual disease, and 2/10 had residual cancer burden scores of 1 (RCB1) (49). The RCB scoring system provides a ranking for the residual disease remaining following neoadjuvant therapy, with RCB0 equating to a pCR and RCB3 indicating extensive tumor burden. The vaccine was determined to be feasible and safe as a neoadjuvant to surgery for patients with TNBC (49).

The third clinical trial evaluated autologous DC vaccine following paclitaxel chemotherapy, prior to surgery and radiation, in patients with stage II/III HER2- BC in a phase 2 trial (NCT00499083). Study results are limited and forthcoming.

A phase 1 trial determined the feasibility and safety of i.t. delivery of CAR-T cells targeting the surface protein hepatocyte growth factor receptor (c-Met) for patients with metastatic BC patients (NCT01837602). c-Met is often over-expressed in several cancers, including BC. c-Met expression served as an eligibility requirement and was evaluated prior to patient enrollment (50). Low grade AEs were reported in some of the patients, including erythema and myalgia, but no CAR-T cell-related AEs above grade 3 were reported (50). CAR mRNA, albeit at low levels, was found in peripheral blood samples 20 minutes and 2 hours post-injection in two and one patients, respectively, but not at later time points (day 1 onward) (50). Necrosis, immune cell infiltration, and hemorrhage were observed within the injected tumor. Overall, the injection was determined to be well-tolerated, safe, and able to induce an i.t. inflammatory response (50).

A phase 1 clinical trial evaluated the safety and feasibility of i.t. delivery of autologous CD1c (BDCA-1)+ myeloid DCs in advanced solid tumors, including TNBC, along with i.t. ipilimumab and avelumab and intravenously (i.v.) delivered nivolumab (NCT03707808). 3/9 of the treated patients had TNBC (48). Only one patient (1/9) experienced a grade 3+ AE, and the most common AEs were local and generalized pruritus (3/9). All three BC patients had progressive disease post-treatment, though one of the patients initially had a mixed response with regression in a treated lesion but the occurrence of additional lesions. Overall, the treatment was demonstrated to be safe and feasible (48).

Other clinical trials delivering cells without posted study results include delivery of the activated, allogeneic Th1-like cells AlloStim sponsored by Immunovative Therapies, Ltd., with a phase 1/2 trial first posted in 2009 for patients with metastatic cancers including BC (NCT00861107) and a phase 1/2 trial first posted in 2010 for patients with stage II-IV cancers including BC (NCT01065441).

#### 3.1.2 Viruses

Six clinical trials utilized viruses either to directly lyse tumor cells or to deliver genes as a delivery vector. A non-replicating adenovirus encoding IL-12 (Ad-RTS-hIL-12) was evaluated in phase 1/2 (NCT02423902) and phase 2 (NCT01703754) clinical trials in patients with locally advanced or metastatic BC and with locally recurrent or metastatic BC, respectively. In the Ad-RTS-hIL-12 system, the expression of IL-12 was inducible via an oral activator ligand, veledimex (51). This platform increased IL-12, IFNγ, and CD8+ T cell infiltration and reduced Tregs within the injected tumor. A reduction in non-injected lesion size was also observed. Furthermore, using immune Response Evaluation Criteria in Solid Tumors (iRECIST) guidelines, 5/9 subjects demonstrated either partial response or stable disease, having a disease control rate of 44% and 22% at week 6 and 12, respectively, post-treatment. Because of the inducible production of IL-12 via veledimex, AEs were controllable, leading to a good safety profile (51).

In a phase 1 clinical trial, the oncolytic herpes simplex virus type 1, HF10, was delivered to patients with cutaneous and/or superficial cancers, including BC (NCT01017185). Initial data demonstrated that the injections were safe and well-tolerated, with mild AEs and rapid viral clearance from the saliva, urine, and blood (52).

In another phase 1 trial, the oncolytic measles virus encoding thyroidal sodium iodide symporter (NIS) (MV-NIS) was delivered once i.t. to evaluate safety and tolerability in patients with metastatic BC (NCT01846091). NIS is a plasma protein which assists in iodide transport. NIS uptake of systemically administrated Tc-99m pertechnetate or I-123 coupled with SPECT-CT imaging provided feedback on viral propagation. Six patients received increasing doses of MV-NIS, with no dose-limiting toxicities observed (53). AEs included grade 2 lymphopenia and fatigue and grade 1 flu-like symptoms. Clinically, at greater than six weeks post-treatment, 4/6 had stable disease, 1/6 had a clinical response, and 1/6 experienced disease progression. The treatment was determined to be safe, with evidence suggesting MV-NIS activity (53).

Other trials with unposted results included i.t. adenoviral-mediated delivery of unregulated IL-12 (NCT00849459) and p53 (NCT00004038) in patients with metastatic BC.

#### 3.1.3 Nucleic acids

In a phase 1, non-randomized, open-label trial, metastatic TNBC patients received three doses of plasmid IL-12 intratumorally followed by electroporation on days 1, 5, and 8 (NCT02531425). Previously, in preclinical studies, plasmid IL-12 both expanded and activated CD8+ TILs and sensitized mice to checkpoint inhibitor therapy (54). These results were shown to be correlated with a higher chemokine CXCR3 gene signature (54). In the clinical trial, four of nine treated patients exhibited higher CD8+ TILs post-treatment, which were associated with increases in the expression of genes associated with T cell activation, cytokine signaling pathways, antigen presentation, and chemotaxis. Additionally, one patient previously unresponsive to aPD-1 therapy demonstrated clinical response to aPD-1 following plasmid IL-12 delivery and electroporation. Overall, the results suggested the treatment to be safe with an increased CXCR3 gene signature prognostic of a positive response (54).

#### 3.1.4 Innate immune agonists

To date, two clinical studies have evaluated an innate immune agonist in BC patients. A safety study was performed with the TLR9 agonist IMO-2125 in patients with refractory solid tumors, including BC (NCT03052205). I.t. injections were given on Days 1, 8, and 15. Fever, chills, and fatigue were the most common AEs. Immunologically, at 24 hours post-treatment, gene expression of IFN gamma, MHC I and II, and type 1 IFN pathways increased within the tumor. Overall, i.t. IMO-2125 was well-tolerated and induced favorable anti-tumor immune responses (55, 56).

The safety and tolerability of i.t. E7766, a STING agonist, was studied in patients with lymphoma and advanced solid tumors, including BC (NCT04144140). The study was completed in 2022, however, no results have been posted.

#### 3.1.5 Bacteria

One completed phase 1 clinical trial tested the feasibility and safety of a single i.t. injection of *Clostridium novyi-NT* spores in patients with advanced solid tumors, including BC, in a 3 + 3 dose escalation study (NCT01924689). Two of the 24 patients had BC; one patient was not evaluable, and the other patient had stable disease (57). Overall, of the 22 evaluable patients, 9 had tumor regression and 19 had stable disease. Treatment promoted tumor-specific T-cell and systemic cytokine responses. However, toxicities were non-negligible, with grade ≥3 AEs comprised of gas gangrene (n=1), abscess limb (n=1), respiratory insufficiency (n=1), rash (n=1), pathologic fracture (n=1), sepsis (n=2), and soft-tissue infection (n=1) (57).

### 3.2 Ongoing clinical trials

As of January 2024, 32 active and/or recruiting clinical trials are pursuing i.t. delivery of therapeutics for BC indications following the same search query as that from the completed clinical trials (**Table 2**, **Figure 2**). Reported updates, if provided, are as follows.

**Table 2:** Ongoing (recruiting and/or active) clinical trials of intratumoral immunotherapies for breast cancer (clinicaltrials.gov, key search terms “breast cancer” AND “intratumoral” OR “intralesional”) as of January 2024.

Intratumoral Intervention	Description	BC Indication	NCT Number	Phase
Antibodies				
pembrolizumab	aPD-1 checkpoint inhibitor; some with mRNA-2752, an mRNA for OX40L, IL-23, and IL-36γ [neoadjuvant]	High Risk DCIS	NCT02872025	0
Cells				
DC1	HER2-primed DCs [neoadjuvant]	HER2+ BC	NCT03387553	0
DC1	HER2, HER3-primed DCs	Early Stage TNBC	NCT05504707	1
DC1	HER2-primed DCs [neoadjuvant]	Stage I-III HER2+ BC	NCT05325632	2
huCART-meso cells	anti-mesothelin immunoreceptor M5 CAR-T cells	Metastatic/Advanced TNBC	NCT05623488	1
Viruses				
ADV/HSK-tk	oncolytic adenovirus encoding herpes simplex virus thymidine kinase; *with stereotactic body radiation therapy and valacyclovir*	Metastatic/Advanced TNBC	NCT03004183	2
BT-001	oncolytic vaccinia virus encoding for aCTLA-4 and GM-CSF	Metastatic/Advanced TNBC	NCT04725331	1/2
CAdVEC	oncolytic adenovirus encoding IL-12 and aPD-L1	HER2+ BC	NCT03740256	1
CF33-hNIS-antiPDL1	chimeric orthopoxvirus (CF33) encoding hNIS and anti-PD-L1	Metastatic TNBC	NCT05081492	1
JX-594	oncolytic vaccinia virus encoding GM-CSF and LacZ and designed to inactivate the thymidine kinase gene; *with cyclophosphamide and avelumab*	Metastatic/Advanced	NCT02630368	1/2
MEM-288	oncolytic adenovirus vector encoding interferon beta (IFNβ) and a CD40-ligand (MEM40)	Metastatic/Advanced TNBC	NCT05076760	1
MV-s-NAP	oncolytic measles virus encoding helicobacter pylori neutrophil activating protein (NAP)	Metastatic Invasive TNBC	NCT04521764	1
R130	oncolytic herpes simplex virus type 1	Advanced	NCT05860374, NCT05961111, NCT05886075	0
T3011	oncolytic herpes simplex viruses encoding IL-12 and aPD-1	Advanced	NCT05602792	1/2
T-VEC	oncolytic herpes virus talimogene laherperepvec [neoadjuvant]	Early Stage	NCT03802604	0
T-VEC	oncolytic herpes virus talimogene laherperepvec [neoadjuvant]	TNBC	NCT02779855	1/2
T-VEC	oncolytic herpes virus talimogene laherperepvec	Metastatic/Advanced HER2-	NCT03554044	1
VSV-IFNβ-NIS (VOYAGER V1™; VV1)	oncolytic Vesicular stomatitis virus (VSV) encoding Interferon-beta (IFNβ) and the sodium iodide symporter (NIS) [neoadjuvant]	Invasive	NCT01042379	2
VV-GMCSF-Lact	vaccinia virus encoding colony-stimulating factor and oncotoxic protein lactaptin genes	Recurrent/Refractory Metastatic	NCT05376527	1
*Nucleic Acids*				
mRNA-2752	mRNA for OX40L, IL-23, and IL-36γ	Metastatic/Advanced TNBC	NCT03739931	1
stimotimagene copolymerplasmid	DNA plasmid in polycationic envelop encoding herpes simplex virus thymiidne kinase and human granulocyte-macrophage colony-stimulating facor; *with ganciclovir*	Advanced	NCT05578820	1
Tavokinogene Telseplasmid	DNA plasmid encoding for human IL-12; *with pembrolizumab*	Inoperable TNBC	NCT03567720	2
TriMix	mRNA encoding CD70, CD40 ligand, constitutive active toll-like receptor 4 [neoadjuvant]	Early Stage	NCT03788083	1
Innate Immune Agonists				
CAN1012	TLR7 agonist	Advanced	NCT05580991	1
CMP-001	virus-like particle encasing a TLR9 agonist [neoadjuvant]	Early Stage	NCT04807192	2
CMP-001	virus-like particle encasing a TLR9 agonist	Metastatic/Advanced	NCT04916002	2
ONM-501	STING agonist	Advanced TNBC	NCT06022029	1
Poly ICLC	TLR3 agonist	Advanced	NCT04116320	1
Bacteria				
*Clostridium novyi-NT*	attenuated anaerobic bacterium	Advanced	NCT03435952	1
SGN1	oncolytic bacterium	Advanced	NCT05103345	1

#### 3.2.1 Cells

In a phase 1/2 clinical, DCs primed with HER2-antigens were i.t. delivered to patients with HER2+ BC (NCT05325632). Results from the phase 1 study indicated that of the 12 patients receiving treatment, three had complete responses, six had partial responses, and three had stable disease. Of these patients, nine had surgery, with six obtaining a pCR. Common AE’s included chills (50%), fatigue (42%), diarrhea (42%), headache (42%), and nausea (42%). Immunologically, the higher dose (100 million cells) had a higher HER2-specific T cell response than the lower dose (50 million cells) six weeks following DC treatment (58).

#### 3.2.2 Checkpoint inhibitors and other antibodies

Intratumoral administration of pembrolizumab is being evaluated in patients with high risk DCIS in the neoadjuvant setting (NCT02872025). One cohort in this phase 1 trial is also receiving an mRNA construct encoding OX40L, IL-23, and IL-36γ (mRNA-2752). Higher immune cell infiltrates correlated with improved prognosis for patients with DCIS, providing the rationale for local pembrolizumab delivery (59). In an interim analysis, the injected doses, even at the smallest amount (2mg), led to increased CD8+ and overall T cell tumor infiltration (59). The overall DCIS volume was not dramatically altered with treatment, and additional anti-tumor responses were not observed, suggesting another agent may be required for tumor reduction (59).

#### 3.2.3 Viruses

The oncolytic adenovirus CAdVEC, which encodes for IL-12 and aPD-L1, was delivered in four metastatic BC patients as part of a phase 1 trial (NCT03740256) (60, 61). A single i.t. injection caused an influx of CD8+ T cells and TME repolarization without significant toxicity (60). Gene set enrichment analysis of tumors pre- and post- treatment indicated antigen processing and presentation enrichment (60). AE’s were either grade 1/2, with fatigue, fever, and injection site pain being the most frequently reported (61). Clinically, when combined with systemic checkpoint inhibitors, CAdVEC caused local and abscopal antitumor responses, with three partial responses observed (60, 61).

T-VEC delivered i.t. in the neoadjuvant setting with paclitaxel/doxorubicin/cyclophosphamide chemotherapy was evaluated in 37 patients with nonmetastatic TNBC in a phase 2 trial (NCT02779855) (62). The most common AEs included pain at the injection site, chills, fever, headache, and fatigue (62). Surgery was utilized for RCB assessment. The primary end point, RCB0 rate, was met (45.9%), and 2-year disease-free rate (overall 89%) had no recurrences in patients with RCB0-1 scores. Immunologically, i.t. immune activation was observed, with CD8+ effector T cell and CD3+CD45RO+ memory T cell i.t. density increased six weeks post-treatment (62).

T-VEC was also delivered i.t. in the adjuvant setting, in combination with chemotherapy or endocrine therapy in advanced/metastatic HER2- breast cancer (NCT03554044). Observed grade 3+ adverse events included neutropenia (5/19), thrombocytopenia (1/19), ulceration at the injection site (1/19), and anemia (1/19) (63). Response rates were obtained for 18/19 patients, with 11/18 obtaining a partial response, 4/18 had stable disease, with the remaining 3/18 progressing. Immunologically, the treatment altered the systemic immune profile (63).

I.t. administered oncolytic herpes simplex viruses encoding IL-12 and aPD-1 (T3011) in patients with advanced solid tumors (NCT05602792) reported an update of no dose limiting toxicities (DLTs) and no systemic viral transmission (64). The chimeric orthopoxvirus (CF33) encoding human NIS and anti-PD-L1 (CF33-hNIS-antiPDL1) was delivered in a phase 1 trial for metastatic TNBC (NCT05081492). Though studies are ongoing, an update at SABCS 2022 indicated no DLTs were reported in the six patients receiving dose levels 1 and 2 (1 and 3x10^5^ pfu, respectively) (65).

#### 3.2.4 Nucleic acids

mRNA-2752, which encodes OX40L, IL-23, and IL-36γ, was evaluated in advanced or metastatic solid cancers, including BC, and lymphoma (NCT03739931). Dose escalation studies revealed a DLT at the 8mg dose (66). Many inflammatory cytokines were upregulated in the tumor and in the plasma following treatment, which also led to T cell infiltration and activation (66).

Tavokinogene Telseplasmid (IT-tavo-EP), a DNA plasmid encoding for hIL-12 followed by electroporation, was delivered to sensitize patients with inoperable locally advanced or metastatic TNBC to pembrolizumab in a phase 2 trial (NCT03567720) (67). Of the 11 patients evaluated, three patients had a partial response, and some untreated tumors regressed (67). The objective response rate (ORR) was 27.3%. 68.8% of patients experienced an AE, with 37.5% being grade ≥3 (67). Findings suggested IT-tavo-EP could improve checkpoint inhibitor therapy in these heavily pretreated patients.

#### 3.2.5 Bacteria

*Clostridium novyi-NT*, an attenuated anaerobic bacterium, was evaluated in advanced BCs and other solid malignancies in a phase 1 trial (NCT03435952) (68). No DLTs were observed, and the injection was determined to be feasible. Though neither of the two partial responses observed were in BC patients, the therapy exhibited anti-tumor potential (68).

## 4 Preclinical studies

Preclinically, i.t immunotherapies such as antibodies, cells, viruses, cytokines, and innate immune agonists have been utilized with varying degrees of success. Furthermore, in an effort to enhance intratumoral retention of immunotherapies after injection, a range of delivery systems have also been developed. The most frequently explored delivery systems include NPs, microspheres, and hydrogels. NPs are ~1-100 nm in diameter whereas microspheres/microparticles range from 1-1000 um in diameter. Hydrogels are three-dimensional polymeric networks encapsulating large amounts of water. The following subsections will highlight preclinical studies investigating each type of immunotherapy both without and with delivery strategies. Each therapeutic delivery highlights the timing of delivery, as well as its unique delivery system, should it contain one. Preclinical studies were obtained from a PubMed search using the terms: “intratumoral” AND “immunotherapy” AND “breast cancer”, as of January 2024.

### 4.1 Checkpoint inhibitors and other monoclonal antibodies

Several preclinical studies have investigated the utility of i.t. delivery of monoclonal antibodies (mAbs) both with and without local retention strategies (**Table 3**).

**Table 3:** Intratumoral antibody therapies in BC preclinical studies.

Immunotherapeutic	Additional Treatment	Delivery Medium	Treatment Frequency	Outcome	Cell Line (Model)	Immuno-competent	Tumor Size or Initial Treatment Timing	Ref.
2E4-PE38	–	–	2	Elimination	66c14 (BALB/c)	Yes	5 days p.i.	(69)
IgG + TNFa + aCD40	–	–	4	Regression	4T1 (BALB/c)	Yes	14 days p.i.	(70)
aCD47 + cGAMP	–	–	1	Delay	E0771 (C57BL/6)	Yes	7 days p.i.	(71)
anti-OX40	cuttlefish ink-based NPs (i.t.), PTT	–	4	Delay	4T1 (BALB/c)	Yes	8 days p.i.	(72)
aCD40 + aPDL1	RT	NDES	1	Regression	4T1 (BALB/c)	Yes	150mm^3^	(73)
aOX40 + aCD40	–	NDES	1	Delay	4T1 (BALB/c)	Yes	130mm^3^	(74)
aPD-1	celecoxib (i.t.)	alginate hydrogel	1	Delay	4T1 (BALB/c)	Yes	7 days p.i.	(75)

#### 4.1.1 Without a local delivery system

Anti-OX40 mAbs were i.t. delivered on day 10 and 11 post-4T1 tumor implantation in a murine model, which directly followed two consecutive days of administration of cuttlefish ink-based NPs, which are natural photothermal agents that have also immune activity (72). Treatment delayed tumor growth, while also inhibiting lung metastasis, reducing splenomegaly, increasing activated IFNγ+ CD4+ and CD8+ splenocytes, and increasing tumor infiltrating CD8+ T cells (72).

Anti-CD47 mAb delivered i.t. in combination with 2′3′-Cyclic GMP-AMP (cGAMP) seven days after tumor implantation led to a complete cure in 5/9 treated E0771 tumor-bearing mice after a single injection. Efficacy was dependent on STING- and type I interferon-responses. The combination treatment enhanced CD8+ T cell immunity and induced anti-tumor immunity (71).

In another study, allogenic immunoglobulin G (IgG) in combination with DC stimuli tumor necrosis factor alpha (TNFα) and aCD40 was delivered twice, two days apart, in two cycles 14-16 days post 4T1 tumor implantation in a mouse model. Treatment induced near complete regression of 4T1 tumors, with 100% survival at day 34 following implantation (70).

In a fourth study, the variable domain of anti-mCD25 was attached to a portion of Pseudomonas exotoxin A (2E4-PE38) and delivered on days 5 and 9 post-tumor implantation in a 66c14 murine BC tumor (69). The treatment increased functional IFNγ+ CD8+ T cells, cDCs, and macrophages, while decreasing Tregs. Cured mice were immune to rechallenge, and the effect was abrogated by CD8+ T cell depletion (69).

#### 4.1.2 With a local delivery system

In an effort to improve therapeutic index and formulate synergistic drug combinations, aPD-1 and celecoxib, a nonsteroidal anti-inflammatory drug, were delivered within an alginate hydrogel injected subcutaneously adjacent to orthotopic 4T1 tumors seven days after tumor inoculation in a murine model (75). The combination within the gel delayed tumor growth and increased survival as well as decreased lung metastases, compared to the either single agent in the gel or the combination in a saline solution (75).

A nanofluidic drug-eluting seed (NDES) locally released the agonist mAbs aOX40 and aCD40 in an orthotopic 4T1 BC murine model to reduce delivery injections and localize treatment (74). The NDES was implanted using the trocar technique, which physicians regularly use to place drain tubes in patients. The NDES released payloads through physical and electrostatic interactions. Delivery of single agents (aOX40 or aCD40 alone) demonstrated slightly delayed tumor growth compared to untreated controls. Both single agents increased i.t. CD4+ and CD8+ T cells, as well as T cells expressing CD134/OX40, which is important for T cell expansion and survival. In the spleen, the percentage of CD8+ T cells secreting IFNγ and granzyme B increased. While the treatments only moderately slowed the growth of 4T1 tumors, the NDES device did demonstrate efficacy retaining the biologic activity in a sustained release system with reduced hepatocellular toxicity compared to systemically treated mice (74). In a follow-up study, aCD40 and aPD-L1 were delivered in the NDES device in larger tumor burdens of 150 mm^3^, with radiotherapy administered for three consecutive days prior to NDES implantation (73). The therapy increased survival and reduced tumor burden in a 4T1 murine model of BC. The antitumoral effects were associated with decreased liver inflammation, increased i.t. CD8+ T cells, higher proliferating Ki67+ CD4+ T cells, and reduced M2 macrophages. Though not directly shown, the dual delivery with RT was suggested to induce systemic antitumoral immunity (73).

### 4.2 Cells

Preclinical local cell delivery for BC treatment has included CAR-T cells, cytokine-induced killer (CIK) cells, fibroblasts, DCs, and xenogeneic mammary glandular cells. To date, no cellular therapy for BC treatment has incorporated a delivery system (**Table 4**).

**Table 4:** Intratumoral cell therapies in BC preclinical studies.

Immunotherapeutic	Additional Treatment	Treatment Frequency	Outcome	Cell Line(s) (Model)	Immuno-competent	Tumor Size or Initial Treatment Timing	Ref.
HER2 CAR-T	–	1	Elimination	BBM1, BT474 (NSG mice)	No	8 days p.i.	(76)
DCs	vector-activated 5-fluorouracil (i.t.)	1	Elimination	CCL-51 (BALB/c)	Yes	100mm^3^	(77)
iPSC-DCs	RT, aPD-L1 (i.p.)	4	Elimination	AT3 (C57BL/6)	Yes	7 days p.i.	(78)
DCs	aHER2 (i.p.)	6	Elimination	TUBO (BALB/c)	Yes	12 days p.i.	(79)
Her2NG CAR-T	–	4	Regression	spontaneous, erbB-2 (F1 heterozygotic Her2NG)	Yes	40mm^3^	(80)
fibroblasts	–	1	Delay	SB-5b (C3H/He)	Yes	0 days p.i.	(81)
XMCs	gemcitabine (i.p.)	1	Delay	4T1 (BALB/c)	Yes	30-50mm^3^	(82)
c-MET CAR-T	cyclophosphamide (i.p.)	4	Delay	BT20, MDA-MB-231, SK-OV-3 (NSG mice)	No	6 weeks p.i.	(50)
CIK cells + cetuximab	–	5	Delay	patient-derived (NSG mice)	No	500mm^3^	(83)
HER3-DC	–	5	Delay	4T1, TUBO (BALB/c)	Yes	7 days p.i.	(84)

HER2-targeted CAR-T cells were delivered intracranially to treat experimental HER2+ BC metastases in the brain. Experimental metastases were established via stereotactic injection of BBM1 and BT474 cell into the brain parenchymas of immunodeficient NOD/scid/γc(-/-) (NSG) mice (76). Local intracranial delivery of HER2 CAR T cells with either a 4-1BB or CD28 costimulatory domain eliminated brain metastases and extended survival past 100 days post tumor implantation, whereas control mice did not survive past 35 days of treatment. Intraventricular delivery of HER2 CAR-T cells with the 4-1BB co-stimulatory domain improved mouse survival compared to intracranial delivery (100% vs 50% at Day 100 post-tumor implantation), and furthermore it could eliminate leptomeningeal and multifocal disease (76).

Another CAR-T cell therapy targeted c-MET, which is expressed by 50% of all BCs (50). *In vivo*, 1.5x10^7^ cells were delivered i.t. in the human xenograft SK-OV-3/luc model in NSG mice, concurrently with cyclophosphamide, on weeks 6, 7, 9, and 11 after tumor inoculation. Compared to controls, the c-MET CAR-T cells delayed tumor growth, which has led to a phase 0 clinical trial (50).

A third CAR-T cell therapy utilized F1 heterozygotic Her2NG offspring from Her2NG transgenic male mice and wild-type FVB/N strain female mice to evaluate CAR-T cells expressing scFvs that bind Her2NG (80). In the i.t. setting, 1.5x10^7^ cells were delivered every two weeks for a total of four doses. Treatment led to regression of tumors, which eventually regrew. Though administered into one tumor locally, CAR-T cells were found in other mammary gland tumors as well as peripheral lymphoid organs.

CIK cells, which share functional characteristics of both T cells and NK cells, confer a unique benefit to treatment, as they do not require antigen for priming (83). CIK cells express FcγRIIIa (CD16a) and, in combination with monoclonal antibodies, can lyse tumor cells. In a patient-derived TNBC model in NSG mice, 1x10^7^ CIK cells were delivered i.t. in combination with the anti-epidermal growth factor receptor (EGFR) mAb cetuximab once daily for five consecutive days when tumors had reached 500mm^3^. Treatment induced a delay in tumor growth as well as increased CD3+ T cell density within the tumor (83).

In another study, fibroblasts were utilized as vaccine carriers to treat BC that had metastasized to the brain (81). Fibroblasts were transfected with DNA from a breast tumor, SB-5b, which was spontaneously derived from a C3H/He mouse (H-2Kb). Fibroblasts additionally expressed MHC class I H-2K^b^-alloantigens and were modified to secrete IL-2, IL-18, or GM-CSF. Mice treated intracranially with IL-2 expressing fibroblasts survived longer than mice in the control or other treatment groups. Splenocytes from these mice demonstrated higher cell lytic capacity as well as higher IFNγ production, demonstrating an increased anti-tumor activity (81).

DCs were also utilized as a vaccine after being transfected with an adenovirus encoding a secretory signal peptide with or without tumor associated antigen (Ad-sig or Ad-sig-TAA) linked to the extracellular domain of CD40 ligand (edcCD40L) (77). They were delivered i.t. in combination with the AdCDIRESE1A vector that encodes for bacterial cytosine deaminase, an enzyme that produces the chemotherapy 5-fluorouracil from 5-fluorocytosine. DCs activated with the Ad-sig-ecdCD40L vector and AdCDIRESE1A vector treatment eliminated tumors when injected i.t. in CCL-51 murine BC, enhancing survival compared to the other treatment groups. This combination treatment further reduced the development of lung metastasis compared to a single agent alone. When the gene for a TAA was incorporated into the adenovirus (rat H2N), and rH2N transgenic mice bearing rH2N+ NT2 breast tumors were treated i.t. with Ad-sig-rH2N/ecdCD40L DCs and the AdCDIRESE1A vector, tumor growth was delayed and survival was extended, as compared to the other treatment groups (77).

A more recent study with DCs evaluated their combination with aHER2 mAb after being pulsed with multiepitope MHC II HER2 peptides (79). In mice bearing HER2+ orthotopic TUBO tumors, DCs delivered i.t. together with intraperitoneal (i.p). aHER2 eliminated tumors and extended survival, compared to the single agents. Mice treated s.c. with this same combination only had a delay in tumor growth. The combination i.t. treatment led to increased IFNγ production from splenocytes and cells isolated from the tumor draining LN. Additionally, higher Th1 cytokines IFN-γ and TNF-α were found in the serum. Increased levels of CD4+ and CD8+ T cells, NK, NKT, and B cells were seen in the tumor. The treatment also led to the regression of untreated distant tumors (79).

A DC vaccine pulsed with HER3 antigen was i.t. delivered to subcutaneous 4T1 murine tumors seven days after tumor implantation (84). Treatments given twice a week for a total of six doses led to a delay in tumor growth and prolonged survival. Flow cytometry analysis revealed that greater numbers of CD4+ and CD8+ T cells per tumor mass had infiltrated the tumor compared to control treatments. Phenotypically, HER3-DC treatment increased the number of intratumoral CD4+ effector and memory cells. In culture, LN lymphocytes from the HER3-DC treated cohort produced higher amounts of IFNγ compared to control. Furthermore, these results were replicated in a HER2+ TUBO murine mammary carcinoma model, when DCs were delivered once weekly for a total of six doses (84).

DCs derived from induced pluripotent stem cells were i.t. delivered in combination with RT in the AT3 murine BC model thirteen days post tumor implantation (78). Three doses of DCs with two doses of RT led to slight tumor regression and prolonged survival, compared to DC treatment alone. Mechanistically, RT helped to direct the DCs to the tdLN and upregulate CD40 expression. The synergistic treatment increased intratumoral stem-like progenitor exhausted CD8+ T cells. In an abscopal tumor model, treatment of the primary tumor lead to tumor growth delay in the abscopal treatment, with a higher CD8+ TIL percentage of CD45+ cells in the distant tumor. Finally, RT and DC treatment sensitized AT-3 murine tumors to aPD-L1. Four DC i.t. injections, three RT treatments, and six aPD-L1 i.p. injections led to long-term, specific, durable cures in 30% of treated mice (78). Another cellular therapy investigated i.t. delivery of xenogeneic mammary glandular cells (XMCs) in combination with gemcitabine (82). Delivering xenogeneic tissue-specific cells was hypothesized both to induce an anti-inflammatory response and to promote cross-reactive antitumor T cells due to shared protein expression between XMCs and cancerous mammary tissue (85). In small 30-50mm^3^ 4T1 mouse tumors, 1x10^6^ XMCs isolated from porcine mammary glands were delivered i.t. once, along with three doses of gemcitabine delivered weekly i.p. The XMC treatment led to tumor growth delay, with higher percentages of necrotic areas and apoptotic cells and fewer Ki67+ proliferating cells. Additionally, i.t. CD4+ and CD8+ T cells and CD56+ NK cells increased, along with a decrease in CD11b+ MDSCs (82).

In summary, cellular therapies in preclinical murine models primarily evaluated efficacy of DCs (77–79, 84) and CAR-T cells (50, 76, 80). No treatment incorporated a local delivery system. No single cell therapy could eliminate tumors without an additional treatment in immunocompetent murine models (**Table 4**). However, with repeated injections and/or additional therapy, tumors could be eliminated in immunocompetent mice (77–79). I.t. delivery outperformed subcutaneous delivery (79), with indication of systemic anti-tumor effects (79, 80).

### 4.3 Viruses

The largest amount of preclinical interest in BC i.t. immunotherapy has been in the area of virus delivery. Oncolytic virus and viral gene therapy were explored in multiple settings. All studies lacked delivery systems as encapsulation of viruses in polymeric particles or hydrogels impairs infectivity (**Table 5**).

**Table 5:** Intratumoral viral therapies in BC preclinical studies.

Immunotherapeutic	Additional Treatment	Treatment Frequency	Outcome	Cell Line (Model)	Immuno-competent	Tumor Size or Initial Treatment Timing	Ref.
Immunotherapy Treatment							
AdCAIL-2	–	1	Elimination	cells derived from spontaneously developed tumors in MMTV-PyMT transgenic mice (FVB/n)	Yes	21 days p.i.	(86)
Ad.mB7-1, Ad.mB7-2 +Ad.IL-2	–	1	Elimination	cells derived from spontaneously developed tumors in MMTV-PyMT transgenic mice (FVB/n)	Yes	75-150mm^3^	(87)
ADV/4-1BBL + ADV/IL-12	–	1	Elimination	JC (BALB/c)	Yes	5x5mm	(88)
CF189	-	1	Regression	MDA-MB-468 (athymic nude mice)	No	100-150mm^3^	(89)
reovirus type 3 Dearing strain	CD3xHER2 bispecific Abs (i.p.)	2	Regression	BT474 (PMBC reconstituted NSG mice)	Yes	34 days post BT474 engraftment	(90)
adenoviral-aCTLA-4	aCD25 (i.p.)	1	Delay	*neu*+ MMC (Her2/neu transgenic mice)	Yes	4x3mm diameter	(91)
Ad.HSK-TK, Ad.GM-CSF, Ad.IL-2	–	1	Delay	4T1 (BALB/c)	Yes	50-100mm^3^	(92)
Ad.IR-E1A/TRAIL + Ad.Flt3L or Ad.GM-CSF	-	1	Delay	C3L5 (C3H)	Yes	100mm^3^	(93)
miR-CVB3-1.1	-	1	Delay	4T1 (BALB/c)	Yes	20mm	(94)
polio immunization	–	1	Delay	E0771 (C57BL/6)	Yes	26 days p.i.	(95)
TPV/Δ66R/mCCL2 and TPV/Δ66R/mIL-2	-	1	Delay	MDA-MB-231 (athymic nude mice)	No	50mm^3^	(96)
rAd.sT expressing sTGFbRIIFc	aPD-1, aCTLA-4 (i.p.)	2	Delay	4T1 (BALB/c)	Yes	7 days p.i.	(97)
SFV/IFNg + Pam3CSK4	–	2	Delay	4T1 (BALB/c)	Yes	4 or 7 days p.i.	(98)
adenovirus encoding P60		3	Delay	LM3 (BALB/c), E0771 (C57BL/6)	Yes	500mm^3^	(99)
AdLyp.sT	aPD-1, aCTLA-4 (i.p.)	3	Delay	4T1 (BALB/c)	Yes	7 days p.i.	(100)
HAd-EphrinA1-Fc + HAd-Flt3L	–	3	Delay	MT1A2 (FVB/n)	Yes	Stage 1: 1 week p.i.; Stage 2: 30-50mm^3^	(101)
RdB/IL-12/DCN	-	3	Delay	4T1 (BALB/c)	Yes	110-120mm^3^	(102)
rV-neuT	–	3	Delay	spontaneous (BALB/c mice transgenic for the rat neu oncogene*)*	Yes	6 weeks post birth	(103)
oncolytic reovirus	aPD-1 (i.p.)	4	Delay	EMT6 (BALB/c)	Yes	6 days p.i.	(104)
Adenovirus encoding Flagrp-170	–	5	Delay	4T1 (BALB/c)	Yes	4-5mm in diameter	(105)
JX-594	aPD-1, aCTLA-4 (i.p.)	8	Delay	spontaneous (MMTV-PyMT transgenic model)	Yes	9 weeks post birth	(106)
rNDV-P05	–	3	None	4T1 (BALB/c)	Yes	7 days p.i.	(107)
Neoadjuvant Treatment							
eCPMV	–	2	Regression	inflammatory (canine)	Yes	not applicable	(108)
eCPMV	–	2-8	Regression	inflammatory (canine)	Yes	not applicable	(109)
Ad.mGM-CSF/mIL-12	4-1BB ligand (i.p.), mIg-mGITRLs (i.p.)	2	n/a	4T1 (BALB/c)	Yes	3-4mm in diameter	(110)

#### 4.3.1 Immunotherapy treatment

##### 4.3.1.1 Oncolytic viruses

Oncolytic adenoviruses encoding the LyP-1 peptide, which binds to the p32 receptor that is highly expressed on BC tumors and has a strong correlation with TNBC, were also engineered to express sTGFbRIIFc, which can inhibit transforming growth factor-β (TGFβ) signaling (100). I.t. delivery of these adenoviruses led to a delay in tumor growth and enhanced aPD-1 and aCTLA-4 checkpoint inhibitor therapy, with a reduction in lung metastases, in 4T1 murine tumors (100).

An oncolytic adenovirus expressing IL-12 and the proteoglycan decorin which can reduce TGFβ immunosuppression (RdB/IL12/DCN), was given every other day thrice to 110-120 mm^3^ 4T1 mouse tumors (102). Treatment delayed tumor growth, increased proinflammatory cytokine expression in tumor tissue, increased i.t. CD8+ T cells, and reduced i.t. TGFβ which correlated with i.t. Treg reduction (102).

An oncolytic adenovirus which expressed a modified receptor for TGFβ (sTGFbRIIFc) to inhibit TGFβ signaling was delivered twice, on days 7 and 9/10 post implantation, in 4T1 murine tumors (97). Treatment downregulated TGFβ target genes and inhibited Th2 cytokine expression, while increasing Th1 cytokines. Additionally, peripheral blood memory T cells and splenic DCs increased, while MDSCs were reduced. Concurrent treatment with the adenovirus improved checkpoint inhibitor therapy (97).

An oncolytic adenovirus was constructed for use in BC cells that allow E1-deleted adenovirus viral replication that was restricted to BC cells via genome modification (inverse repeat adenovirus vectors) (93). Additionally, the E1A and TNF-related apoptosis inducing ligand (TRAIL) proteins were inserted into the vector to support viral replication and induce tumor apoptosis (Ad.IR-E1A/TRAIL). Researchers combined the Ad.IR-E1A/TRAIL oncolytic virus with an adenoviral vector carrying Flt3L or GM-CSF gene to induce a vaccination-type response. Different combinations of 1x10^9^ pfu adenoviruses were delivered once to C3L5 murine tumors, and the most effective combination were Ad/IR-E1A/TRAIL + Ad.Flt3L, which outperformed combinations with Ad.GM-CSF and single therapies (93).

Another study attempted to enhance the efficacy of checkpoint inhibitors through the i.t. administration of an oncolytic vaccinia virus expressing GM-CSF (JX-594) in MMTV-PyMT transgenic mice which spontaneously develop tumors (106). JX-594 had previously been thought to serve as an *in situ* vaccine due to its ability to elicit an anti-tumor immune response and its synergy with aPD-1 in Renca tumors. JX-594 was delivered i.t. (1x10^7^ pfu per tumor) eight times in the course of eight weeks, with systemic delivery of aPD-1 and aCTLA-4. Tumor growth was delayed and survival extended (106 days compared to 87 days), with an increase in i.t. CD4+, CD8+, CD8+ ICOS+, and CD8+ Granzyme B+ T cells (106).

The chimeric parapoxvirus CF189 was constructed as a therapeutic against TNBC (89). Different doses (10^3^, 10^4^, 10^5^ PFU) were evaluated in MDA-MB-468 tumors in athymic nude mice, with regression observed in the lowest dose (10^3^ PFU) when tumors 100-150mm^3^ in size receive one injection of the virus. The highest dose (10^5^ PFU) in the injected tumor caused a slight tumor regression in the contralateral un-injected tumor, demonstrating the induction of systemic anti-tumor immunity from a local treatment (89).

A coxsackievirus B3 virus was modified with a targeting sequence miR145/143 to prevent normal tissue targeting and reduce toxicity (94). 4T1 murine tumors 20mm in size were treated with 1x10^6^ PFU. Treatment delayed tumor growth and reduced metastasis in correlation with viral propagation and F4/80+, CD4+, and CD8+ immune cell infiltration, with reduced toxicity compared to wild type coxsackievirus B3 (94).

A tanapoxvirus (TPV) was engineered to express monocyte chemoattractant protein (CCL2) or IL-2 (TPV/D66R/mCCL2 and TPV/D66R/mIL-2) as a safer oncolytic virus with selectivity for tumor cells (96). One injection of 5x10^6^ PFU in ~50mm^3^ MDA-MB-468 tumors in athymic nude mice led to tumor growth delay for both TPV/D66R/mCCL2 and TPV/D66R/mIL-2, with increased areas of necrosis and immune cell infiltration (96).

To increase efficacy of systemically delivered CD3-bispecific antibodies, an oncolytic, replication-competent reovirus type 3 Dearing strain was delivered twice 34-days after BT474 implantation in peripheral blood mononuclear cells (PBMC) reconstituted NSG mice (90). Three i.p. injections of CD3xHER2 bispecific antibodies were administered following the i.t. reoviral treatment. The reoviral treatment itself led to tumoral regression and, in combination with the bispecific Abs, led to additional regression (90).

Another oncolytic reovirus was used to treat palpable EMT6 murine tumors (104). Four injections of reovirus on days 6, 9, 12, and 14 post tumor inoculation at 5x10^8^ PFU delayed tumor growth. Additional treatment with aPD-1 cured ~70% of treated mice at 110 days post tumor implantation. Immunologically, both reovirus and combination treatments increased splenic CD4+ and CD8+ T cells and T_EM_ cells. Reoviral treatment upregulated i.t. Tregs, but aPD-1 reversed this phenomenon. The reoviral treatment and aPD-1 demonstrated systemic memory through re-challenge rejection as well as increased systemic anti-tumor IFNγ-producing CD8+ T cells (104).

Not all i.t. delivered oncolytic viruses are effective, especially as compared to systemic treatment. A recombinant Newcastle disease virus (NDV) virus strain 5 (rNDV-P05) was delivered to 4T1murine tumors thrice, spaced a week apart (107). No effect was observed, though the systemic administration of the virus did yield an anti-tumor effect (107).

##### 4.3.1.2 Viral gene vectors

Non-oncolytic viruses employed as gene vectors have also been well studied. An adenovirus vector carrying human IL-2 gene (AdCAIL-2) was delivered i.t. to tumor cells derived from tumors of MMTV-PyMT transgenic mice which, as noted above, spontaneously develop tumors (86). Direct injection with 5x10^8^ pfu of AdCAIL-2 led to complete tumor regression in 8/9 mice within 3-4 weeks post-treatment. Antitumor activity was contingent on delivery location, as a subcutaneous injection of AdCAIL-2 on the opposite flank of the tumor did not lead to any delay in tumor growth. Tumor regression was associated with long-term anti-tumor immunity, as demonstrated by a lack of tumor growth in rechallenge studies (86).

A human adenovirus vector encoding EphrinA1-Fc, a ligand of tyrosine kinase receptor EphA2 overexpressed in BC which can inhibit tumor progression, and one expressing Flt3L were evaluated in small (30-50mm^3^) EphA2 overexpressing MT1A2 tumors in a murine model (101). Ad.EphrinA1-Fc led to tumor growth delay, which was further reduced by incorporating Ad.Flt3L (101).

To inflame the immune environment, immunostimulatory proteins and ligands αCTLA-4, αCD127, αCD3, IL-15, LIGHT, mda-7, and CD80 have been incorporated into adenoviruses (91). However, none displayed anti-tumor activity when delivered into 4x3mm HER2/neu positive mouse mammary carcinoma (MMC) tumors. Tumor delay was observed when the mAb aCD25 was delivered with i.t. adenoviral-aCTLA-4. Survival was extended ~6 days in the combination group compared to aCD25 mAb treatment alone (91).

An adenovirus encoding B7-1 (Ad.mB7-1) and B7-2 (Ad.mB7-2), ligands for T cell receptor CD28, were coupled with adenoviral-IL-2 delivery in PyMT and neu tumors to induce complete tumor regression after a single i.t. injection in a mouse model (87). In PyMT tumors, 7/15 tumors completely regressed following Ad.mB7-2/hIL-2 treatment, compared to 4/15 in the Ad.mB7-2 treatment group. PyMT-specific cytotoxic T cell activity was observed following these treatments via an *in vitro* cytotoxic T lymphocyte (CTL) assay. In neu tumors, 13/20 completely regressed in Ad.mB7-1/hIL-2, 9/15 in mB7-2/IL-2, demonstrating tumor dependent differences in response (87).

Three adenoviral constructs containing genes for herpes simplex virus thymidine kinase (HSV-TK), which confers cytotoxicity to the nucleoside analog ganciclovir by phosphorylating ganciclovir, GM-CSF, and IL-2, respectively, were engineered for immune stimulation as well as concurrent treatment with ganciclovir (92). In 50-100mm^3^ 4T1 murine tumors, 1x10^10^ adenovirus particles injected at three different tumor sites. The greatest tumor delay involved the triple combination of GM-CSF, IL-2 and HSV-TK adenoviruses, which reduced lung metastases and correlated with an increase in lymphocyte infiltration. To evaluate how neoadjuvant treatment impacted metastasis, tumors were excised three days after adenoviral treatment, but no effect on metastasis was observed between surgery and no surgery groups 15 days after surgery (92).

Liver metastasis in the JC mouse mammary model were treated using an adenoviral-mediated delivery of the 4-1BB ligand (ADV/4-1BBL) co-delivered with an adenoviral delivery of IL-12 gene (ADV/IL-12) (88). 4-1BBL is a co-stimulatory receptor expressed on the surface of activated T cells. Liver metastases were treated once i.t. with ADV/4-1BBL and ADV/IL-12 in 5x5mm tumors. 78% of treated mice survived at least 150 days post tumor implantation, which was primarily mediated by CD4+ T cells. 5/7 of these mice rejected a tumor re-challenge. This ADV-mediated, i.t. treatment was compared to a systemically administered 4-1BBL mAb plus i.t. ADV/IL-12, which caused tumor rejection in 87% of mice. However, unlike the i.t. ADV/4-1BBL, the effect was mediated by CD8+ T cells, and only 3/8 of the mice rejected re-challenge (88).

An innate immune agonist, Flagrp-170, was delivered via adenoviral vector five times, given every other day, in 4-5mm diameter 4T1 murine tumors (105). Flagrp-170 contains an NF-κB-stimulating sequence from flagellin incorporated into the Grp-170 molecular chaperone and has demonstrated greater anti-tumor activity than flagellin alone. Treatment caused i.t. Th1-associated cytokine gene signature upregulation (IFNγ, IL-12, GM-CSF). Splenocytes and cells from the draining LN produced more IFNγ and IL-2 compared to untreated controls. The treatment delayed tumor growth, the effect of which was abrogated by inhibition of GM-CSF (105).

Expression of the transcription factor Foxp3 in both Tregs and in BC cells was targeted with a cell penetrating peptide P60 that binds to a region of Foxp3 encoded in an adenoviral vector (99). When HER2+ LM3 and TNBC E0771 mouse tumors reached a burden of 500 mm^3^, the vector was delivered once every three days for a total of three treatments, leading to tumor growth delay and decreased lung metastases. Treatment led to a reduction of Tregs in the tumor but not the spleen (99).

Recombinant vaccinia virus encoding for the neu oncogene was tested in neu transgenic BALB/c mice, and the stage of cancer, number of injections, and delivery route were all optimized (103). Mice survived longer when they received three injections (1x10^8^ pfu/injection), were treated earlier, and were injected in the intramammary gland (intratumoral) compared to subcutaneously. Furthermore, intramammary gland injections led to higher anti-neu antibody titers and more cytotoxic splenocytes compared to subcutaneous vaccination (103).

A replication-deficient Semliki Forest virus (SFV) vector, expressing IFNγ (SFV/IFNγ) was utilized as a safer viral mediated delivery option and ideal for macrophage polarization, as SFVs do not infect human or murine macrophages but do infect other cells (98). When 4T1 murine tumors became palpable, SFV vectors were injected i.t., with a second dose administered six days later. Treatment delayed tumor growth, associated with an increase in CD4+ and CD8+ T cells and a decrease in Tregs, CD11b+ MDSCs and CD206+ M2 macrophages in the tumor (98).

##### 4.3.1.3 Other virus application

Poliovirus delivery was utilized to leverage pre-existing anti-polio immunity induced from childhood vaccination to inflame the TME (95). Prior to E0771 tumor implantation, mice received prime and boost polio vaccination. 26 days post tumor implantation, 1x10^7^ PFU of mRIPO, a version of a live-attenuated poliovirus type 1 vaccine adapted for mice, was delivered once. mRIPO treatment extended survival (50% at 40 days p.t. compared to less than 30 days for other treatment and control groups) and increased CD4+ T cells and eosinophils. CD4+ and CD8+ T cells displayed a higher functional status, indicated by reduced T cell exhaustion markers. UV-inactivated polio virus was also evaluated. 18 days post tumor implantation, the inactivated virus was delivered twice, six days apart, which also delayed tumor growth (95).

#### 4.3.2 Neoadjuvant treatment

Two studies explicitly utilized vectors to induce an adaptive immune response prior to surgical resection. In a canine study of inflammatory BC, a rare type of the disease, empty cowpea mosaic virus (eCPMV) immunotherapy was delivered i.t. weekly (0.2–0.4 mg per dose) for a minimum of two injections to evaluate the safety and efficacy of treatment (108). eCPMV was not intended to infect cells but rather to serve as an innate immune agonist, similar to TLR and STING agonists. Five canine patients were treated, while five were provided firocoxib/cyclophosphamide/toceranib therapy as the control arm. After 14 days following the first injection, tumors had measurably decreased, allowing for surgery in two of the five patients who previously would not have been eligible. Phenotypically, the Treg+/CD8+ T cell ratio in peripheral blood decreased in the eCPMV treatment arm. eCPMV also altered the TME, with higher neutrophil counts and lower Ki-67 expression, as indicated by immunostaining. Combining eCPMV with the standard of care improved the mean survival compared to standard of care alone (134 vs. 67 days) (108).

In a follow-up study, a transcriptomal analysis of canine patients with inflammatory BC again treated with eCPMV revealed an increase in neutrophil recruitment and activation transcriptional profile (109). Tumors in this study also decreased in all six treated patients, which allowed three patients to undergo surgical treatment (109).

In another neoadjuvant study, an adenovirus encoding GM-CSF and IL-12 was delivered i.t. twice every three days alongside systemic 4-1BB ligand (two treatments, each three days apart) and mIg-mGITRLs (8 treatments, each three days apart) in 4T1 mouse tumors (110). mIg-mGITRLs are monoclonal antibody homodimers against glucocorticoid-induced tumor necrosis factor receptor family-related protein, which is primarily expressed on Tregs, and binding is thought to eliminate Tregs through ADCC. After 9 days following the first i.t. treatment, tumors were resected. The triple combination increased survival (>125 days in 65% of mice) and decreased tumor metastases. Mechanistically, within the spleen, the cytolytic activity of T cells increased and Treg function decreased (110).

Overall, viral delivery has been explored in both neoadjuvant and non-neoadjuvant settings, without delivery systems (**Table 5**). Most treatments did not have an additional systemic therapy, but those that did often incorporated checkpoint inhibitors (97, 100, 104, 106), reflecting the ability of certain viruses to sensitize the TME to these already FDA-approved therapies. Three therapies could eliminate tumors with a single injection (86–88), though most viral therapies caused only a delay in tumor growth even with repeated injections (**Table 5**). I.t. treatment could lead to a reduction in lung metastasis (92, 100) and growth delay in untreated, contralateral tumors (89). Route of delivery impacted treatment, as i.t. delivery outperformed subcutaneous delivery when tested in two studies (86, 103). In the neoadjuvant setting, treatment allowed patients in canine studies to undergo surgery when previously surgery had not been an option (108, 109), providing additional rationale to pursue i.t. viral therapy in the neoadjuvant setting.

### 4.4 Nucleic acids

Plasmid DNA encoding cytokines have been delivered i.t. to alter the TME of breast tumors in several preclinical studies. Because plasmids are negatively charged macromolecules, they require delivery assistance, through either electroporation or lipid nanoparticle formulation in order to enter BC cells (**Table 6**).

**Table 6:** Intratumoral nucleic acid therapies in BC preclinical studies.

Immunotherapeutic	Delivery Medium/Strategy	Treatment Frequency	Outcome	Cell Line (Model)	Immuno-competent	Tumor Size or Initial Treatment Timing	Ref.
IL-15/IL-15Rα DNA	electroporation	3	Elimination	4T1-luc2 (BALB/c)	Yes	30-90mm^3^	(111)
pcDNA3.1-IL-7	electroporation	1	Delay	TM40D (BALB/c)	Yes	5 days p.i.	(112)
pcDNA3.1-IL-10 trap	lipid-protamine-DNA NPs	3	Delay	4T1 (BALB/c)	Yes	9 days p.i.	(113)

Plasmid encoding for the inflammatory cytokine IL-15 and IL-15 receptor alpha (IL-15Rα), which stabilized and improves the bioactivity of IL-15, was delivered three times with electroporation within one week to 4T1-luc mammary mouse tumors (111). Tumors in 25% (2/8) of the mice were eliminated, which was associated with an increase in memory T cells, particularly CD8+ T_EM_ and T_CM_ and CD4+ T_CM_). Cured mice rejected tumor rechallenge (111).

The human cDNA of the cytokine IL-7, which can promote T cell survival, was encoded in a plasmid (pcDNA3.1-IL-7) and injected once i.t. followed by electric pulses in a TM40D mouse graft model of BC five days after tumor implantation (112). Treatment delayed tumor growth, the effect of which was dependent on CD8+ T cells. Serum IFNγ and splenocyte CTL cytotoxicity increased (112).

Rather than delivery of an immunostimulatory cytokine, another strategy to modulate the TME is to remove an immunosuppressive cytokine. A gene encoding an interleukin-10 (IL-10) trap protein was delivered i.t. in 4T1 murine tumors, as IL-10 had been correlated with reduced overall survival in patients with TNBC (113). The trap gene was loaded into lipid-protamine-DNA NPs and injected thrice i.t., leading to a delay in tumor growth and 80% survival at 26 days post-treatment as compared to 0% survival at this timepoint in the control groups (n=8-10 for groups). TNFα and IFNγ i.t. gene expression had increased post-treatment compared to controls (113).

### 4.5 Cytokines and chemokines

As observed above, localized cytokines have the potential to modulate the TME from immunosuppressive to immunostimulatory. Several potent cytokine and chemokine therapies have been evaluated in preclinical BC studies with or without a supportive delivery vehicle (**Table 7**).

**Table 7:** Intratumoral cytokine and chemokine therapies in BC preclinical studies.

Immuno-therapeutic	Additional Treatment	Delivery Medium	Treatment Frequency	Outcome	Cell Line (Model)	Immuno-competent	Tumor Size or Initial Treatment Timing	Ref.
Immunotherapy Treatment								
Flt3L + TLR3/CD40 agonists	RT	–	5	Delay	AT-3 (C57BL/6)	Yes	7 days p.i.	(114)
Flt3L + TLR3/CD40 agonists	RT	–	9	Delay	AT-3 (C57BL/6), 4T1 (BALB/c)	Yes	16 (AT-3) or 5 (4T1) days p.i.	(115)
GM-CSF	aPD-1	–	15	Delay	cells derived from spontaneously developed tumors in MMTV-PyMT transgenic mice (FVB/n)	Yes	50mm^3^	(116)
IL-12 + TNFα	–	PLA microspheres	1	Elimination	MT-901 (BALB/c)	Yes	11 days p.i.	(117)
IL-12 + TNFα + IL-18	–	PLA microspheres	1	Delay	4T1 (BALB/c)	Yes	8-10mm^2^	(118)
XCL-1	doxorubicin in NPs (i.v.)	sodium-alginate hydrogel	1	Delay	4T1-luc (BALB/c)	Yes	100mm^3^	(119)
Neoadjuvant Treatment								
Flt3L + TLR3/CD40 agonists	RT	–	5	Delay	4T1 (BALB/c), E0771 (C57BL/6), AT3 (C57BL/6)	Yes	2 days p.i.	(120)
IL-12	–	chitosan	3	Elimination	4T1 (BALB/c)	Yes	6 days p.i.	(121)
CCL21	–	hydron	1	Delay	Cl-66 (BALB/c)	Yes	60mm^3^	(122)

#### 4.5.1 Immunotherapy treatment

##### 4.5.1.1 Without a local delivery system

The cytokine GM-CSF was delivered at low and high doses i.t. in immunologically “cold” PyMT mouse tumors to evaluate the effects of dosing on tumor vasculature normalization and hypoxia (116). Delivered thrice per week over three weeks, low dose GM-CSF reduced tumor hypoxia while normalizing tumor vasculature. However, a five-week treatment with low dose GM-CSF did not affect tumor growth. When used as a pre- and concurrent treatment with aPD-1, low dose (5ng) but not high dose GM-CSF (100ng) led to tumor growth delay. Mechanistically, low dose GM-CSF reduced the inflammatory transcriptional profile of TAMs (116).

##### 4.5.1.2 With a local delivery system

The proinflammatory cytokines IL-12 and TNFα were encapsulated in polylactic acid (PLA) microspheres and injected once i.t. in the weakly immunogenic MT-901 murine model of BC eleven days after tumor implantation (117). Following treatment, ~70% mice were disease-free, which was higher than IL-12 and GM-CSF-loaded microspheres (~40%) and either of the single cytokines alone-loaded microspheres (~2%). T cells derived from both the draining LN and spleen demonstrated higher activity, as measured through IFNγ production. The treatment also led to an increase in i.t. polymorphonuclear cells and CD8+ T cells, measurable five days post-treatment. When compared to treatment with surgery in rechallenge studies, all mice treated with the IL-12 and TNFα microspheres rejected the tumor, whereas only 20% of mice who had received surgery remained tumor-free. Local injection in a sustained delivery medium could eliminate tumors and establish immune memory (117).

A second study with PLA microspheres utilized a triple combination of cytokines, IL-12, IL-18, and TNFα (118). Mice bearing 4T1 tumors received a single i.t. injection of IL-12/IL-18/TNFα microspheres or dual-combination cytokines. The IL-12/TNFα combination led to the most pronounced delay in tumor growth, least metastatic lung nodules, and greatest survival. Surprisingly, the addition of IL-18 to the IL-12/TNFa combination reduced antitumor activity. Antitumor effects were contingent on both CD8+ T cells as well as NK cells. Local cytokine delivery enhanced CD8+ T cell numbers and reduced CD4+CD25+ Tregs in the tumor draining LN (118).

A sodium-alginate hydrogel was constructed to deliver lymphotactin, XCL-1, i.t. in combination with systemic delivery of doxorubicin-loaded poly(lactic acid-co-glycolic acid) (PLGA) NPs coated with cell membranes (119). The NPs would target the tumor, at which point the chemotherapy would induce immunogenic cell death (ICD) and the XCL-1 chemokines would recruit DCs into the tumor to enhance tumor antigen cross presentation. The sodium alginate was delivered as a solution and crosslinked *in situ* due to the presence of calcium ions in the tumor. The gel+XCL-1 was delivered once i.t. in 4T1-luc murine tumors the day after a series of three doxorubicin treatments and delayed tumor growth in the treated and untreated tumors, as well as increased survival at 30 days post-treatment. Furthermore, compared to doxorubicin-NP treatment alone, the XCL-1 treatment increased the homing of CD11c+ DCs to the tumor (119).

#### 4.5.2 Neoadjuvant treatment

##### 4.5.2.1 Without a local delivery system

A neoadjuvant study pursued a combinatorial approach with i.t. and peritumoral immunotherapy and local radiotherapy prior to breast tumor resection in a mouse model (120). The cytokine Flt3L was i.t. injected daily over a period of five days, followed by 9 Gy radiotherapy the next day, and the TLR3 agonist poly(I:C) and aCD40 agonist delivered the following day peritumorally (termed “ISIM” treatment). Six days after poly(I:C)/aCD40 administration, tumors were resected. In 4T1-luc tumors, treatment delayed metastatic tumor growth and increased median survival (34 days vs. 26 days for the control). Furthermore, 6 days after poly(I:C)/aCD40, higher antigen-specific CD8+ were observed in the blood and lungs, along with higher percentages of effector T cells. As determined through depletion studies, prolonged survival was dependent on both CD4+ and CD8+ T cells. Batf3-/- mice with AT-3 tumors did not benefit from the triple combination, the improvement in survival seen in wild type mice was completely abrogated, though it could be moderately rescued by delivering bone-marrow–derived CD103^+^ DCs. The improvement in survival was demonstrated to be dependent on T cell egress from the LNs and immune activation in the tumor draining LN. Addition of systemic neoadjuvant aPD-L1 enhanced median survival (44 days vs. 35 days without aPD-L1) in 4T1-luc tumors. Additionally, increasing radiotherapy treatments to three and performing surgery only one day after poly(I:C)/aCD40 treatment both independently enhanced survival in mice bearing orthotopic 4T1-luc tumors (120). Prior studies with this ISIM treatment in the primary treatment setting demonstrated that it increased frequencies of activated, effector CD4+ and CD8+ T cells in AT-3 murine tumors (114) and decreased PMN-MSDCs in AT-3 and 4T1 BC murine tumors (115).

##### 4.5.2.2 With a local delivery system

The chemokine CCL21, which recruits T cells, NK cells, and DCs, was formulated in Hydron, a hydrophilic polymeric hydrogel, and delivered to cl−66 mouse mammary tumors (122). Local delivery increased total tumor CD8+ T cell and NK cell numbers and enhanced i.t. DC recruitment. As a neoadjuvant, CCL21-Hydron was implanted four days prior to resection. CCL21-Hydron and resection improved overall survival (>70% at 100 days) compared to any other treatment group and delayed tumor growth in a re-challenge model (122).

In another local neoadjuvant study, our group co-formulated the cytokine IL-12 in the naturally occurring polysaccharide chitosan and delivered the co-formulation i.t. in 4T1 murine tumors three times, three days prior to tumor resection (121). Survival was extended (67% of mice were tumor-free >80 days post-resection), and lung metastases were reduced. Upon rechallenge, mice that had eliminated 4T1 tumors did not experience tumor recurrence. Splenocytes demonstrated a higher lytic capacity and enhanced tumor-specific responses, all without measurable systemic toxicity (121).

### 4.6 Innate immune agonists

Research utilizing innate immune agonists to modulate the immune landscape intratumorally, has largely focused on TLR and STING agonists, with and without delivery systems (**Table 8**). Treatments with other agonists, such as RLR agonists, and neoadjuvant studies have also been performed. Innate immune agonist delivery represents the second largest group studied in preclinical studies.

**Table 8:** Intratumoral innate immune agonist therapies in BC preclinical studies.

Immuno-therapeutic	Additional Treatment	Delivery Medium	Treatment Frequency	Outcome	Cell Line (Model)	Immuno-competent	Tumor Size or Initial Treatment Timing	Ref.
Immunotherapy Treatment								
superantigen	–	–	4	Elimination	4T1 (BALB/c)	Yes	7 days p.i.	(123)
prodrug vinyl-phosphonate CDNs	–	–	3	Regression	4T1 (BALB/c)	Yes	60-70mm^3^	(124)
cGAMP	–	–	2	Delay	4T1 (BALB/)	Yes	5 days p.i.	(125)
influenza vaccine	–	–	3	Delay	4T1 (BALB/c)	Yes	10-25mm^2^	(126)
RR-CDA	DC101 (aVEGF), aPD-1	–	6	Delay	spontaneous (MMTV-PyMT mice)	Yes	9 weeks post birth	(127)
poly(I:C) + apoptin gene	–	–	7	Delay	4T1 (BALB/c)	Yes	not provided	(128)
CpG	catalase (i.t.), ^131^I (i.t.), RT	alginate hydrogel	1	Elimination	4T1 (BALB/c)	Yes	60mm^3^	(129)
imiquimod	IcG (i.t.), PTT, cyclo-phosphamide (i.p.)	chitosan-hyaluronic acid hydrogel	1	Elimination	4T1 (BALB/c)	Yes	100mm^3^	(130)
imiquimod	aPD-1 and aCTLA-4 (i.p.); cryoablation	PLGA-PEG-PLGA hydrogel	1	Elimination	EMT-6 (BALB/c)	Yes	8 days p.i.	(131)
imiquimod + aPD-L1	doxorubicin (i.t.)	alginate hydrogel	1	Elimination	4T1-luc (BALB/c)	Yes	2 weeks p.i.	(132)
CpG	doxorubicin (i.t.), polydopamine (i.t.), PTT	hyaluronic acid hydrogel	1	Regression	4T1 (BALB/c)	Yes	100mm^3^	(133)
poly(I:C)	RT	polyethyl-enimine nanoplex	6	Regression	TS/A (BALB/c)	Yes	10 days p.i.	(134)
CpG	SP_II_ nanorods (i.t.), PTT	alginate hydrogel	1	Delay	4T1 (BALB/c)	Yes	100mm^3^	(135)
CpG	^131^I (i.t.), RT	elastin-like polypeptides	1	Delay	4T1 (BALB/c)	Yes	100mm3	(136)
Lipoamino-glycosides	–	vesicle self-assembly	2	Delay	4T1 (BALB/c)	Yes	100-200mm^3^	(137)
CpG	–	Fe_3_O_4_ NPs	3	Delay	4T1 (BALB/c)	Yes	50mm^3^	(138)
imiquimod	doxorubicin micelles (i.v.)	acetylated chondroitin sulfateproto-porphyrin	5	Delay	4T1 (BALB/c)	Yes	50mm^3^	(139)
resiquimod	–	platelet membrane-coated NPs	5	Delay	4T1 (BALB/c)	Yes	5 days p.i.	(140)
poly(I:C)	–	polyethyl-enimine nanoplex	6	Delay	4T1 (BALB/c)	Yes	13 days p.i.	(141)
RIG-I agonists	–	extracellular vesicles	6	Delay	4T1 (BALB/c)	Yes	10 days p.i.	(142)
Outer Membrane Vesicles	–	EGFR-targeted vesicles	11	None	4T1 (BALB/c)	Yes	0.7mm diameter	(143)
Neoadjuvant Treatment								
CDN	IL-2 (i.p.), aPD-1 (i.p.)	–	3	Elimination	4T1-luc (BALB/c), E0771 (C57BL/6)	Yes	100 or 200 mm^3^	(144)
3M-052	–	–	1	Delay	4T1 (BALB/c)	Yes	5 days p.i.	(145)

#### 4.6.1 Immunotherapy treatment

##### 4.6.1.1 Without a local delivery system

In the metastatic 4T1 mouse model, the STING agonist cGAMP was delivered i.t. to increase the type I interferon response locally (125). After 16-24 hours, injection led to an i.t. influx of TILs and CD45^+^ CD11b^mid^ Ly6C^+^ F4/80^+^ macrophages, which demonstrated phagocytic activity and produced the proinflammatory cytokine TNFα and chemokines Cxcl10 and Cxcl11. Two treatments of cGAMP at days 5 and 10 after tumor implantation yielded a modest but statistically significant delay in tumor growth, which was dependent on CD8+ T cells, as demonstrated through a CD8+ depletion study. The authors admitted a need for an appropriate delivery system for cGAMP, as cGAMP was likely not present for a sufficient amount of time to assess its full anti-tumor effects (125).

The STING agonist RR-CDA, a synthetic cyclic dinucleotide (CDN), was injected six times i.t. into spontaneously developing mammary tumor MMTV-PyMT mice 9 weeks post-birth (127). The i.t. treatment was combined with systemically delivered VEGF-R2 mAb (DC101) and aPD-1 mAb. The RR-CDA treatment alone delayed tumor growth, but both local and abscopal tumor control was enhanced with the triple combination treatment. The triple combination increased i.t. CD8+ T cell infiltration, decreased the density of the tumor vasculature, reduced lung metastases, and increased overall survival, which posed a strong rationale for STING therapy and antiangiogenic treatment in BC (127).

A novel STING agonist of vinylphosphonate-based CDNs was engineered to elicit a more potent immune response than other STING agonists evaluated in clinical trials, such as ADU-S100 (124). Additionally, masking the negative charge of the CDN by synthesizing it into a prodrug, enhanced potency, both *in vivo* and *in vitro*. When 4T1 murine tumors were ~65mm^3^, three i.t. deliveries, three days apart, of the highest concentration of prodrug led to tumor regression but not elimination, and this cohort had higher anti-tumoral CD8+ T cell levels compared to the vehicle treated group. Overall, prodrugs outperformed their parent compound, resulting in greater tumor growth delay (124).

Injection of the TLR3 agonist poly(I:C) was co-administered alongside a plasmid encoding the viral protein apoptin which induces apoptosis in tumor cells (128). Treatment of 4T1 murine tumors with the apoptin plasmid and poly(I:C) lead to significant growth delays at day 33 post-treatment, which was suggested to be due to induction of apoptosis, as analyzed through TUNEL assay. Locally, higher numbers of CD4+ and CD8+ infiltrated into the tumor, as analyzed via immunohistochemistry. The local treatment led to systemic increases of CD4+ and CD8+ T cell counts and the Th1 cytokines IFN-γ and IL-2, concurrent with a decrease in the Th2 cytokines IL-4 and IL-10 levels, as compared to other treatment and control groups (128).

Another novel agent was explored to modulate the local tumor immune environment, functioning like an innate immune agonist by non-specifically inflaming the TME. A tumor-targeted superantigen was constructed using the third loop of transforming growth factor α (TGFαL3) and staphylococcal enterotoxin type B (123). Seven days post 4T1 murine tumor implantation (20mm^3^), the superantigen was administered i.t. every other day for a total of four treatments. 5/14 (37.5%) of the treated tumors were eliminated and survived 6 months post-treatment, which outshone the systemically treated superantigen cohort (12.5% survival rate). The effect was correlated with an increase in IFNγ and TNFα production from splenocytes, a decrease in proliferation Ki-67 and micro vascularization CD31 levels, and an increase in tumor necrosis (123).

The influenza vaccine was delivered i.t. to induce local inflammation in a 4T1 mouse model (126). When tumors reached 10-25 mm^2^, the influenza vaccine was delivered thrice on consecutive days, which delayed tumor growth compared to a PBS control. Additional vaccinations (seven total) did not cause additional growth delay. Prior flu vaccination did not impair the anti-tumor response. Mechanistically, the 3x influenza vaccine reshaped the tumor immune microenvironment toward a Th1 profile, with increased IL-4 levels. Furthermore, the vaccine was demonstrated to work through its binding ability to sialic acid residues (126).

##### 4.6.1.2 With a local delivery system

Local delivery systems have served to retain and release TLR agonists and RLR agonists preclinically. The innate immune agonist Poly(I:C), which binds to TLR3, MDA5, and RIG-I receptors, was complexed with the polymer polyethylenimine (PEI) and delivered i.t. in a 4T1 mouse model (141). The PEI-complexed Poly(I:C), called BO-112, was administered twice weekly, for a total of six doses, which led to 4T1 growth inhibition (141). In a follow-up study in TS/A tumors, the same treatment was combined with three doses of radiotherapy (RT) (134). The treatment synergized with RT, causing tumor regression in the primary tumor and tumor growth delay in an untreated abscopal tumor. In a bilateral tumor treatment scheme in which only one tumor was treated with both BO-112 and RT and the other was treated with RT alone, both tumors regressed, with over 20% survival at 150 days p.t. (134).

Several studies explored local delivery of the TLR7 agonist imiquimod. In the first study, imiquimod was encapsulated in a temperature responsive hydrogel composed of the naturally occurring polymers chitosan and hyaluronic acid, along with indocyanine green (IcG), for a combined immunostimulatory therapy with photothermal therapy (PTT) (130). Administering the imiquimod-IcG gel and applying laser therapy led to 4T1 murine tumor growth suppression in 8/10 mice. Greater suppression was induced with a low-dose systemic administration of the chemotherapeutic cyclophosphamide administered on day 0 and 7 post-laser treatment, with 10/12 mice experiencing growth suppression in this group. Additionally, the growth of untreated bilateral tumors was also delayed in both of these treatment groups, as compared to controls (130).

In a second study, imiquimod was delivered within an alginate hydrogel to localize treatment in a mouse model (132). Orthotopic 4T1-fLuc tumors were treated two weeks after tumor implantation with the alginate gel, co-formulated with doxorubicin, imiquimod, and aPD-L1. After 25 days post-treatment, no tumor nor metastases were observable, and all mice (10/10) survived at least 120 days post-treatment. Notably, when these therapeutics were delivered i.p., they had no effect on extending mouse survival, highlighting the importance of the delivery system and route (132).

In a third mouse study, imiquimod was loaded into acetylated chondroitin sulfateprotoporphyrin micelles (ACP-R837) and delivered five times at three-day intervals in 4T1 tumors (139). The treatment increased CD8+ T cell density and proinflammatory cytokines TNFα, IL-6, IL-1b, and IFNγ, while decreasing the anti-inflammatory cytokine IL-10. APC-R837 delayed tumor growth without displaying toxicity and extended survival. Mechanistically, it activated the death receptor signaling pathway, as indicated with an increase in caspase-8 expression. Furthermore, combination with systemically delivered micelles containing the chemotherapeutic doxorubicin caused the largest tumor apoptotic areas and lower Ki67 proliferation marker expression (139).

In a fourth imiquimod study, the agonist was formulated in a PLGA- poly(ethylene glycol)(PEG)-PLGA thermoresponsive hydrogel (131). In a bilateral EMT-6 murine tumor model, the imiquimod-gel was injected intratumorally in a single tumor eight days after tumor implantation. With a triple combination treatment of this imiquimod-gel, cryoablation, and systemically delivered aPD-1 and aCTLA-4, 1/18 mice had a 90-day, long-term survival with treated and untreated tumor elimination, demonstrating induction of an abscopal response (131).

Additionally, the TLR7/8 agonist resiquimod (R848) was encapsulated in platelet membrane-coated NPs (140). Five i.t. treatments with the NPs given five days after tumor implantation delayed the growth of 4T1 mouse tumors, decreased metastatic lung nodules, and enhanced survival with a median progression free survival of 23 days, which was 14 days longer than that of the control group (140).

Other studies pursued the TLR9 agonist unmethylated cytosine-phosphate-guanine (CpG) oligodeoxynucleotides, either alone or in combination with another therapy. In one study, CpG was loaded into 3-aminopropyltriethoxysilane-modified Fe_3_O_4_ NPs (FeNPs) (138). CpG-FeNPs were i.t. injected on days 7, 10, and 13 after tumor implantation in a mouse model, and delayed 4T1 tumor growth, with an inhibitory rate of 69%, and reduced lung metastases. Additionally, splenocytes displayed higher lytic activity against 4T1 cells than those from control groups (138).

CpG was combined with the chemotherapeutic doxorubicin and photothermal agent polydopamine (PDA) within a thiolated hyaluronic acid hydrogel (133). Orthotopic 4T1 mouse tumors were injected with the CpG/PDA/doxorubicin hydrogels and, 12-hours after injection, irradiated with a 780-nm NIR laser. One week following treatment, the tumors were irradiated again. Mice treated with the CpG/PDA and CpG/PDA/doxorubicin combinations had the highest levels of serum TNFα and IL-6, as well as splenic CD8+ T cells. At 12 days post-treatment, tumors from the mice treated with the CpG/PDA/doxorubicin and PTT had regressed the most. Finally, in a bilateral tumor model with one tumor treated with local CpG/PDA hydrogel plus PTT along with three treatments of systemic aPD-L1, the growth of the untreated bilateral tumor was delayed, as compared to control groups. This effect was only statistically significant with the addition of aPD-L1 as treatment with the local CpG/PDA hydrogel plus PTT alone did not lead to a statistical difference in anti-tumor rejection (133).

CpG and semiconducting polymer (SP_II_) nanorods were delivered i.t. in a responsive alginate hydrogel (135). Two hours post-injection, 4T1 murine tumors were treated with PTT. After 24 hours, TUNEL staining and Ki-67 staining of the treated tumor demonstrated higher levels of apoptosis and lower levels of cell proliferation, respectively. In a bilateral metastatic model, treatment with CpG/SP_II_/PTT therapy significantly reduced the growth of the untreated distant tumor and had no observable spontaneous metastatic lung nodules. Within the tumor, markers of immunogenic cell death [calreticulin and high-mobility group box 1 protein (HMGB1)] were increased, and the serum proinflammatory cytokines IL-6, TNFα, and IFNγ also were upregulated, as compared to other treatment groups. Finally, higher levels of mature DCs (CD11c^+^CD80^+^CD86^+^) in the tumor draining LNs and CD8+ cytotoxic T cells in untreated distant tumors were recorded (135).

Another in-situ forming alginate hydrogel locally delivered CpG and catalase labeled with ^131^I radioisotope to localize the effects of RT (129). When tumors had reached ~60 mm^3^, hydrogel treatment with concurrent RT in a subcutaneous 4T1 mouse model eliminated all tumors, with all mice (8/8) surviving 60 days post-treatment, compared to other controls which all succumbed to disease within less than a month post-treatment. In an orthotopic 4T1-fLuc model, 16 days-post-tumor inoculation, mice were treated once locally with the CpG/^131^I-Cat/alginate gel, followed by four systemic injections of aCTLA-4. After 90 days, 75% of mice were surviving without any sign of metastasis (129).

A final study of CpG also combined local brachytherapy, this time within thermoresponsive elastin-like polypeptides (ELPs) (136). When injected i.t. within the ELP delivery system in mice, CpG was still present 18 days later, and a high dose (100ug) led to a statistically significant delay in growth and reduction of lung metastases at 13 days post-treatment. Incorporating localized ^131^I brachytherapy led to further delay in tumor growth, extended survival, and reduced lung metastases (136).

Another innate immune pathway, RIG-I, leads to type I interferon production following 5’ triphosphorylated RNA activation. Because immunomodulatory 5’ triphosphorylated RNA (immRNA) are quickly degraded *in vivo*, they were loaded into extracellular vesicles (EVs) derived from red blood cells (RBCs) and delivered i.t. in 4T1 murine tumors (142). I.t. immRNA delivery in RBC-derived EVs six times every three days delayed tumor growth, while increasing the pro-inflammatory and decreasing the anti-inflammatory cytokine gene signatures. Furthermore, immRNA delivery led to an increase in apoptosis, as measured by TUNEL staining. A similar treatment with human CA1a breast tumors in NSG mice also led to a decrease in tumor growth, accompanied by an increase in i.t. neutrophils, macrophages, and DCs. A second RLR agonist, 3p-125b-ASO, also was engineered to silence the oncogene miR-125b, and i.t. delivery of 3p-125b-ASO in RBC-derived EVs following the same schedule as immRNA RBC-derived EVs also led to a reduction in tumor growth, increase in i.t. neutrophils, macrophages, DCs, NK cells, and T cells, increase in i.t. pro-inflammatory cytokines (TNFα, IL-6, IL-12p40), and increase in apoptosis (142).

A novel innate immune agonist was formulating using lipoaminoglycosides, with the polar headgroups being either tobramycin or kanamycin, which can stimulate cellular immunity (137). These lipoaminoglycosides self-assembled into vesicles. In the 4T1 murine model, vesicles were injected into 100-200mm^3^ tumors twice weekly. The treatment reduced tumor weight on day 19 post-treatment (137).

The inherent property of outer membrane vesicles, lipid-bilayer particles excreted from bacteria, to activate TLR ligands was leveraged as an immunotherapeutic and additionally engineered to display an anti-EGFR scFV on the surface (143). Delivered daily 11 times in 4T1 murine tumors once they reached an average diameter of 0.7 mm, the treatment had no effect on tumor growth at the 10ug dose level. However, an i.p. dose of 25ug led to almost complete tumor regression, which was correlated with an increase in necrosis, i.t. M1 macrophage polarization, and T and NK cell presence (143). This effect was a rare instance in which systemic injection outperformed a local delivery.

#### 4.6.2 Neoadjuvant

I.t. delivery of innate immune agonists was pursued in a neoadjuvant setting, without delivery systems. In a first study, the TLR7/8 agonist 3M-052 was delivered i.t. seven days prior to resection in a 4T1 murine model (145). Resected tumors had higher levels of immune cells (B cells, CD4+ and CD8+ T cells, NK cells, macrophages), and this neoadjuvant treatment extended survival, with a median of 20 days, as compared to the control of 10 days post-resection. Depletion of CD8+ T cells and inhibition of Type I IFN signaling reversed the metastasis-free survival benefit of the neoadjuvant treatment. Interestingly, when neoadjuvant aPD-1 was delivered systemically three times in combination with i.t. 3M-052 given once seven days prior to surgery, no improvement in survival or in i.t. immune cell infiltration was observed compared to the same treatment without aPD-1 (145).

I.t. CDN STING agonists were explored along with combinations of extended half-life IL-2 and aPD-1 delivered systemically (144). CDNs were delivered every three days for a total of three times, followed by surgery three days after the last administration, in 4T1-Luc murine tumors. Of the mice whose tumors were treated beginning at 100 mm^3^ (n=10), long-term survival was 60% at 90 days post-treatment. Of the mice whose tumors were treated beginning at 200 mm^3^ (n=10), long-term survival was slightly reduced to 30% at 90 days post-treatment. Additionally, neoadjuvant treatment (90% survival) was demonstrated to be superior to adjuvant treatment (10% survival) at 100 days post-tumor implantation. Immunologically, i.t. CDN and systemic IL-2 plus aPD-1 increased both i.t. and lung CD8+ T cells and effector CD4+ T cells. Depletion studies highlighted the importance of NK cells on therapeutic efficacy, which did not rely on CD8+ and CD4+ cells and neutrophils. NK cells were activated by the treatment, as shown by an increase in granzyme B, perforin, TNFα, IFNγ, IL-2, CD69, Ki67, and PD-1 expression. The systemic upregulation of IL-2 and type I interferons supported the activation of NK cells (144).

In summary, innate immune agonist immunotherapy was the second most pursued type of i.t. delivered immunotherapy within preclinical BC studies. Due to their small size, these agonists could greatly benefit from delivery systems for localization and long-term, sustained release combined with a reduction in treatment number. Hydrogels in particular necessitated only one i.t. injection (129–133, 135). Most innate immune agonists on their own did not induce a strong anti-tumor response as a monotherapy, and combination with chemotherapy, checkpoint inhibitors, PTT, and/or RT were required for robust anti-tumor responses (**Table 8**).

### 4.7 Bacteria

One preclinical study pursued i.t. delivery of a genetically modified *Salmonella* strain expressing a methioninase in nude mice bearing MDA-MB-231 xenografts (146). *Salmonella* had demonstrated preferential accumulation in the tumor in a previous study. Toxicity concerns were reduced by genetically engineering a strain that did not produce endotoxins, VNP20009, as determined by prior studies. As this strain alone could not eliminate tumors, it was additionally engineered to overexpress a methioninase to reduce tumor methionine levels, which can prevent mitosis, cause cell-cycle arrest, and induce apoptosis. I.t. delivery of this bacteria in 60-100 mm^3^ tumors led to tumor growth delay. Treated tumors underwent a liquefaction necrotic process. Furthermore, bacteria were predominately found in the tumor rather than the lung, liver, or kidney at ratios between 500–62,000:1. Finally, methionine was reduced in tumor cells but not systemically (146). The safety and feasibility of the treatment is being evaluated in a phase 1 clinical trial (NCT05103345).

## 5 Key findings

The first major finding of this review is the increasing interest in i.t. delivery of BC immunotherapies. In the past two decades, the number of clinical trials increased steadily (**Figure 2F**). From 2004-2013, 11 clinical trials started, whereas a decade later from 2014-2023, 37 clinical trials began. Immunotherapy itself is strengthening its foothold as a primary therapeutic modality as well as the future of cancer treatment. Annually, a staggering 4 million patients worldwide are eligible for an immunotherapy, according to the CRI (147). Within BC, the impact of immunotherapies is just now being felt. Pembrolizumab was first FDA-approved for locally recurrent unresectable or metastatic PD-L1 positive TNBC in November 2020 and since then has also been approved for high-risk, early-stage TNBC (148). With the rising interest, more i.t. immunotherapies will likely be evaluated in BC.

The second major finding is the proven safety record of i.t. delivered immunotherapies in BC. Results from clinical studies conclude that i.t. immunotherapy is technically feasible and well-tolerated with a low toxicity profile (48–50, 54–56, 149). This feasibility and safety profile indicate that intratumoral injection is a relevant delivery method for BC treatment.

The third major finding is the evidence of both local and systemic efficacy, with induced immune changes, following i.t. immunotherapy. Clinical anti-tumor responses were noted in the following clinical trials: NCT02423902, NCT01703754 (51), NCT01846091 (53), NCT03740256 (60, 61), NCT02779855 (62), and NCT03567720 (67). Numerous preclinical studies across a range of immunotherapies have demonstrated elimination of treated tumors (69, 76–79, 86–88, 111, 117, 121, 123, 129–132, 144). Additionally, a major feature of i.t. immunotherapy is its potential to induce robust systemic immunity capable of controlling distant, untreated lesions. Anti-tumor effects in un-injected, abscopal tumors were observed with the adenovirus Ad-RTA-hIL-12 (51), and adenovirus CAdVEC (60, 61) in clinical studies. Preclinical treatments with antibodies, cells, viruses, cytokines, and innate immune agonists reduced or eliminated metastases (72, 75–77, 92, 94, 99, 100, 110, 118, 120, 121, 127, 129, 132, 135, 136, 138, 140, 145) and induced systemic anti-tumor effect in untreated tumors (79, 119, 130, 131, 133–135). Furthermore, i.t. therapies have both initiated T cell infiltration, as demonstrated in the following clinical trials: NCT02061332 (47), NCT01837602 (50), NCT02423902, NCT01703754 (51), NCT03740256 (60, 61), NCT02779855 (62), NCT02531425 (54), NCT03739931 (66), and NCT02872025 (59). Most, if not all, preclinical studies also demonstrated a skewing toward an anti-tumor immune response intratumorally. The potential of i.t. immunotherapy – treat locally, act systemically – promises great potential for BC patients.

## 6 Next steps

First, a major trend and a significant opportunity is the movement of immunotherapies, including i.t. immunotherapies toward neoadjuvant and earlier stage treatments in BC patients. Immunotherapies are more likely to be effective in BC patients with lower disease burden and intact immune systems. With respect to neoadjuvant BC immunotherapy, currently, high risk patients receive chemotherapy prior to surgery. However, recurrence rates are still high, and side effects detrimentally affect patients’ quality of life. Immunotherapy promises to boost tumor specific immunity that eliminates residual cancer cells after surgery and prevents recurrence. Promising results from the clinical trials NCT02018458 (49), NCT02061332 (47), and NCT02779855 (62) and from the preclinical studies (108, 109, 120–122, 144, 145) encourage more consideration for neoadjuvant i.t. immunotherapy studies in further preclinical research and in developing clinical trials.

Second, the potential to find pCRs following neoadjuvant i.t. immunotherapy in BC is a major opportunity. pCRs may serve as a primary endpoint rather than recurrence which may take a decade or longer in BC patients for targets to be reached. It is expected that increasing numbers of BC trials will utilize neoadjuvant treatments, including i.t. immunotherapies, and measure pathological responses at the time of lumpectomy as the primary efficacy outcome.

**Figure 3:**
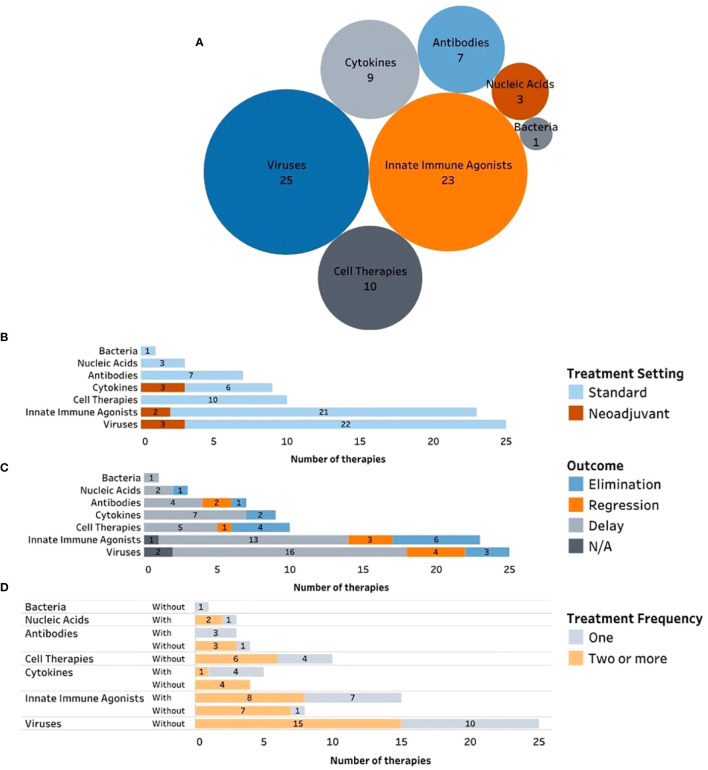
Preclinical intratumoral immunotherapies in breast cancer divided by **(A)** drug modality, **(B)** treatment setting, **(C)** outcome, and **(D)** treatment frequency.

Third, the type of immunotherapy delivered must be given additional consideration. For example, pembrolizumab given i.t. in high-risk DCIS did not decrease tumor volume in a phase 1 clinical trial (59). It is possible that inflaming the immune environment prior to or concurrent with checkpoint inhibitor therapy may synergize with checkpoint inhibitors to produce a better anti-tumor response. This combination treatment was noted in a metastatic TNBC patient receiving plasmid IL-12, whose tumor became sensitized to aPD-1 (54). Additionally, evaluation of preclinical studies did not note a clear trend for efficacy based on immunotherapeutic agent alone (**Figure 3**), which suggests that other factors (timing, frequency, delivery medium) also strongly impact treatment outcome. Currently, viral therapies dominate both clinical and preclinical research, but many of the competing platforms look promising.

Fourth, a delivery system for non-infectious agents should become a trend for i.t. therapy. Developing and incorporating delivery medium into intratumoral treatment can positively impact clinical treatment by decreasing the frequency of i.t. injections, as more numerous injections can decrease patient compliance, increase healthcare burden, and increase local pain associated with injections. Furthermore, delivery mediums can localize the therapeutic locally for extended periods of time, thereby increasing local therapeutic concentration and reducing systemic AEs. Currently evaluated delivery media primarily include hydrogels, microparticles, and NPs (**Figure 3**). Of these, hydrogels require fewer delivery injections than the others and potentially offer a long-term release profile. Furthermore, the tumor microenvironment is extremely dense, and saline-based delivery leaks from the injection site and returns via the needle track. Hydrogels can resist this higher-pressure environment and can be locally retained for extended periods of time, presenting an ideal delivery medium for i.t. therapies.

Overall, as the fields of immunotherapy and i.t. delivery continue to grow in BC, studies should continue to pursue treatment in earlier stages and carefully consider appropriate immunotherapeutics and delivery media to optimize patient treatment outcome.

## Author contributions

SM: Conceptualization, Data curation, Formal analysis, Funding acquisition, Investigation, Methodology, Project administration, Resources, Software, Supervision, Validation, Visualization, Writing – original draft, Writing – review & editing. YA: Conceptualization, Supervision, Writing – review & editing, Investigation. AS: Data curation, Formal analysis, Investigation, Validation, Visualization, Writing – review & editing. SU: Data curation, Formal analysis, Investigation, Validation, Visualization, Writing – review & editing. DZ: Conceptualization, Project administration, Resources, Supervision, Writing – original draft, Writing – review & editing.

## Glossary

**Table:**

aCTLA-4	anti-cytotoxic T lymphocyte-associated antigen 4
AE	adverse event
ALR	AIM2-like receptor
aPD-1	anti-programmed cell death protein 1
aPD-L1	anti-programmed death-ligand 1
BC	breast cancer
CAR	chimeric antigen receptor CDN, cyclic dinucleotide
cGAMP	2’3’-Cyclic GMP-AMP
CIK	Cytokine-Induced Killer
CCL2	monocyte chemoattractant protein
CLR	C-type lectin receptor
CpG	cytosine-phosphate-guanine
CRI	Cancer Research Institute
CTL	cytotoxic T lymphocyte
c-MET	hepatocyte growth factor receptor
DAMP	damage-associated molecular pattern
DC	dendritic cell
DCIS	ductal carcinoma in situ
DLT	dose limiting toxicity
eCPMV	empty cowpea mosaic virus
EGFR	epidermal growth factor receptor
ELP	elastin-like polypeptide
EV	extracellular vesicles
G-CSF	granulocyte colony-stimulating factor
GITR	glucocorticoid-induced tumor necrosis factor receptor family-related protein
GM-CSF	granulocyte-macrophage colony-stimulating factor
HER2	human epidermal growth factor receptor 2
HMGB1	high mobility group box
HSV-TK	herpes simplex virus thymidine kinase
ICD	immunogenic cell death
IcG	indocyanine green
Ig	immunoglobulin
IL	interleukin
IO	Immuno-Oncology
i.p.	intraperitoneal
i.t.	intratumoral
IFN	interferon
immRNA	immunomodulatory 5’ triphosphorylated RNA
LN	lymph node
mAb	monoclonal antibody
MDSC	myeloid-derived suppressor cell
MMC	mouse mammary carcinoma
NDES	nanofluidic drug-eluting seed
NDV	Newcastle disease virus
NIS	sodium iodide symporter
NLR	NOD-like receptor
NK	natural killer
NP	nanoparticle
NSG	NOD/scid/γc(-/-)
ORR	overall response rate
PAMP	pathogen-associated molecular pattern
PBMC	peripheral blood mononuclear cells
pCR	pathological complete response
PD-1	programmed cell death protein 1
PD-L1	programmed death-ligand 1
PEG	poly(ethylene glycol)
PEI	polyethylenimine
PDA	polydopamine
PLA	poly-lactic-acid
PLGA	poly(lactic acid-co-glycolic acid)
PRR	pattern recognition receptor
PTT	photothermal therapy
STING	stimulator of interferon genes
SP_II_	semiconducting polymer
RBC	red blood cell
RCB	residual cancer burden
RECIST	response evaluation criteria in solid tumors
RIG-I	retinoic acid-inducible gene I
RLR	retionic acid-inducible-gene-I-like receptor
RT	radiotherapy
SEER	Surveillance, Epidemiology, and End Results
SFV	Semliki Forest virus
TAA	tumor associated antigen
TAM	tumor associated macrophage
TGFβ	transforming growth factor-β
TIL	tumor infiltrating lymphocyte
TIM-3	T-cell immunoglobulin and mucin domain 3
TLR	toll-like receptor
TME	tumor microenvironment
TNBC	triple negative breast cancer
TNF	tumor necrosis factor
TPV	tanapoxvirus
TRAIL	tumor necrosis factor (TNF)–related apoptosis inducing ligand
Treg	regulatory T cells
T-VEC	talimogene laherparepvec
VEGF	vascular endothelial growth factor
XCL-1	lymphotactin
XMC	xenogeneic mammary glandular cell.
